# Global burden, trends and inequalities of maternal hypertensive disorders among reproductive-age women of advanced maternal age, 1990–2021: a population-based study

**DOI:** 10.3389/fgwh.2025.1513909

**Published:** 2025-03-06

**Authors:** Xuanyu Zhao, Weimin Kong, Yan Jiang, Feng Sui

**Affiliations:** ^1^ Department of Maternal Intensive Care Unit, Beijing Obstetrics and Gynecology Hospital, Capital Medical University, Beijing Maternal and Child Health Care Hospital, Beijing, China; ^2^ Department of Gynecology, Beijing Obstetrics and Gynecology Hospital, Capital Medical University, Beijing Maternal and Child Health Care Hospital, Beijing, China

**Keywords:** maternal hypertensive disorders, global burden, advanced maternal age, health inequality, joinpoint regression

## Abstract

**Background:**

Maternal hypertensive disorders (MHD) are leading causes of maternal morbidity and mortality worldwide, particularly among reproductive-age women of advanced maternal age (AMA), representing a significant global public health challenge.

**Objective:**

This study aimed to analyze the global trends, inequalities, and disparities in the burden of MHD among reproductive-age AMA women from 1990 to 2021.

**Methods:**

We conducted a population-based study using data from the Global Burden of Disease (GBD) 2021 study, covering 204 countries and territories. The study included women aged 35–49 years with hypertensive disorders during pregnancy. We assessed age-standardized incidence rate (ASIR) and age-standardized death rate (ASDR) of MHD among reproductive-age AMA women. Temporal trends were evaluated using joinpoint regression analysis, while health inequalities were measured using the concentration index and the slope index of inequality (SII).

**Results:**

Between 1990 and 2021, the global ASIR of MHD decreased from 568.10 (95% UI: 412.06–738.55) to 491.49 (95% UI: 368.78–619.84) per 100,000 population (AAPC: −0.46%, 95% CI: −0.54% to −0.38%), and ASDR declined from 2.57 (95% UI: 2.23–2.97) to 1.44 (95% UI: 1.19–1.76) per 100,000 population (AAPC: −1.83%, 95% CI: −1.99% to −1.67%). Substantial disparities persisted across socio-demographic index (SDI) regions, with high and high-middle SDI regions showing increasing incidence trends (AAPC: 2.36% and 1.45%, respectively). The slope index of inequality (SII) for ASIR improved from −3,052.73 (95% CI: −3,329.55 to −2,775.91) to −1,209.36 (95% CI: −1,393.12 to −1,025.61) per 100,000 women, while the SII for ASDR decreased from −11.29 (95% CI: −12.38 to −10.20) to −3.66 (95% CI: −4.13 to −3.20) deaths per 100,000 women. The concentration index for ASIR showed slight improvement (from −0.46 to −0.34), while ASDR inequality marginally worsened (from −0.62 to −0.66).

**Conclusion:**

Despite overall declines in MHD burden, significant disparities persist, particularly in low SDI regions. These findings highlight the need for targeted public health interventions to reduce inequalities, improve healthcare access, and enhance maternal outcomes for reproductive-age AMA women globally.

## Introduction

Maternal hypertensive disorders (MHD) are leading causes of maternal and perinatal morbidity and mortality worldwide (1, 2).These disorders, including gestational hypertension, preeclampsia, and eclampsia, have significant implications for both maternal and neonatal health (3–7). An increasing trend of delayed childbearing has led to a notable rise in pregnancies among women of advanced maternal age (AMA), typically defined as age ≥35 years (8).This shift in maternal demographics has impacted the epidemiology of MHD, necessitating a deeper understanding of their burden and trends among reproductive-age AMA women. Previous clinical studies and meta-analyses have demonstrated that AMA is associated with an increased risk of hypertensive disorders during pregnancy. A comprehensive systematic review and meta-analysis by Lean et al. observed that advanced maternal age was associated with an increased risk of hypertensive disorders of pregnancy and other adverse pregnancy outcomes (9). This risk increases progressively with maternal age, with women aged ≥40 years showing even higher risk (10). Recent epidemiological data from the United States shows substantial variations in the prevalence of maternal hypertensive disorders across different age groups, with consistently higher rates among women aged ≥35 years (11). This pattern has also been observed in large-scale population studies in other regions, such as China, where AMA was identified as an independent risk factor for hypertensive disorders during pregnancy (12). This demographic shift, combined with the potentially higher risk profile of AMA pregnancies, makes understanding the global burden of hypertensive disorders in reproductive-age AMA women particularly important for public health planning and healthcare resource allocation.

These hypertensive disorders can lead to severe maternal complications such as stroke, organ failure, and maternal death, as well as adverse neonatal outcomes including preterm birth and low birth weight (10, 13–15). Despite advancements in maternal healthcare and the management of hypertensive disorders, comprehensive global and regional data on the burden and trends of MHD among reproductive-age AMA women remain limited. Existing clinical guidelines often lack specific recommendations for reproductive-age AMA women, and there is a scarcity of population-based data on the inequalities and trends of these disorders across different regions and socioeconomic groups (9, 16). As the number of pregnancies at advanced maternal age continues to rise globally, understanding the burden, trends, and disparities of MHD among this population is increasingly urgent.

Addressing these knowledge gaps is crucial for informing public health interventions and updating clinical guidelines to better manage and mitigate the risks associated with hypertensive disorders in reproductive-age AMA pregnancies. Significant disparities in maternal outcomes related to socioeconomic factors, healthcare access, and regional inequalities need to be systematically explored to develop targeted health policies aimed at reducing preventable maternal morbidity and mortality. Given the unique challenges faced by reproductive-age AMA women, tailored interventions are essential to improve maternal outcomes and reduce health inequities.

The present study aims to examine the global, regional, and national burden, temporal trends, and inequalities in MHD among reproductive-age women of advanced maternal age from 1990 to 2021. We also aim to explore the impact of social determinants, regional differences, and healthcare development on the prevalence and outcomes of MHD in this population. By providing comprehensive and up-to-date data, this study seeks to fill critical gaps in knowledge, ultimately contributing to the improvement of maternal health outcomes and the reduction of disparities for reproductive-age AMA women worldwide.

## Methods

### Data sources and study population

This study utilized data from the Global Burden of Disease (GBD) 2021 study, which provides comprehensive health metrics for 204 countries and territories from 1990 to 2021 (17). We focused on data specific to reproductive-age women of advanced maternal age (35–49 years) with hypertensive disorders in pregnancy. MHD were defined according to the International Classification of Diseases codes: 642–642.9 in ICD-9 and O10–O16.9 in ICD-10, encompassing chronic hypertension, gestational hypertension, preeclampsia, and eclampsia (18). We categorized each country and territory into one of five Socio-demographic Index (SDI) quintiles based on their SDI values (19). The SDI is a composite measure reflecting development status, incorporating income per capita, average educational attainment, and total fertility rate. This classification facilitated the analysis of trends in MHD incidence and mortality across varying levels of socioeconomic development.

The GBD 2021 study employs rigorous methodologies for data collection and processing, as described in detail elsewhere (20). The study synthesizes a large and growing number of data input sources including surveys, censuses, vital statistics, and other health-related data sources. These data sources and detailed methodological information are publicly accessible through the Global Health Data Exchange (GHDx) Sources Tool (http://ghdx.healthdata.org/gbd-2021/sources). This interactive tool allows users to access specific methodological details for different GBD components, causes, risks, and locations.

### Disease burden indicators

We extracted age-standardized incidence and mortality rates as the primary metrics for assessing the burden of MHD. To calculate these rates for the 35–49 age group, we employed the direct standardization method using the GBD 2021 standard population as the reference (21). Age-specific incidence and mortality rates were obtained from our study population and weighted by the corresponding proportion of the GBD 2021 standard population for each age group (22). This approach ensured comparability across different populations by accounting for variations in age distribution. The final age-standardized rates were calculated by multiplying the age-specific rates by the standard population weights and summing the results across the relevant age groups.

### Statistical analysis

We conducted a descriptive analysis of the age-standardized incidence and mortality rates across regions and countries. To assess temporal trends from 1990 to 2021, we performed joinpoint regression analysis to calculate the average annual percentage change (AAPC) and 95% confidence intervals (CIs) for each metric (23). Trends were considered significant if the 95% CI of the AAPC did not include zero. Trends were classified as increasing (both CI limits positive), decreasing (both CI limits negative), or stable (CI includes zero).

### Health inequality analysis

To assess inequalities in the distribution of MHD across countries, we employed two complementary measures: the concentration index and the slope index of inequality (SII).

The concentration index was calculated using the Lorenz curve approach to evaluate the unequal distribution of incidence and mortality rates across countries ranked by their gross domestic product (GDP) per capita (24). The concentration index ranges from −1 to 1, with negative values indicating a concentration of the health outcome among poorer countries and positive values indicating a concentration among wealthier countries. A value of 0 represents perfect equality.

For the SII, we modeled the age-standardized mortality rates against a relative social position scale based on GDP per capita using weighted least squares regression to account for heteroskedasticity (25). The SII represents the absolute difference in the health outcome between the hypothetical countries at the bottom and top of the socioeconomic spectrum. Both inequality measures were calculated for each year from 1990 to 2021 to assess changes in health inequalities over time.

### Software and reporting guidelines

All statistical analyses were performed using the Health Equity Assessment Toolkit from the World Health Organization (WHO), R version 4.4.1, and Stata version 16.0. We adhered to the Guidelines for Accurate and Transparent Health Estimates Reporting (GATHER) (26).

## Results

### Global trends in ASIR and ASDR

**Figure 1:**
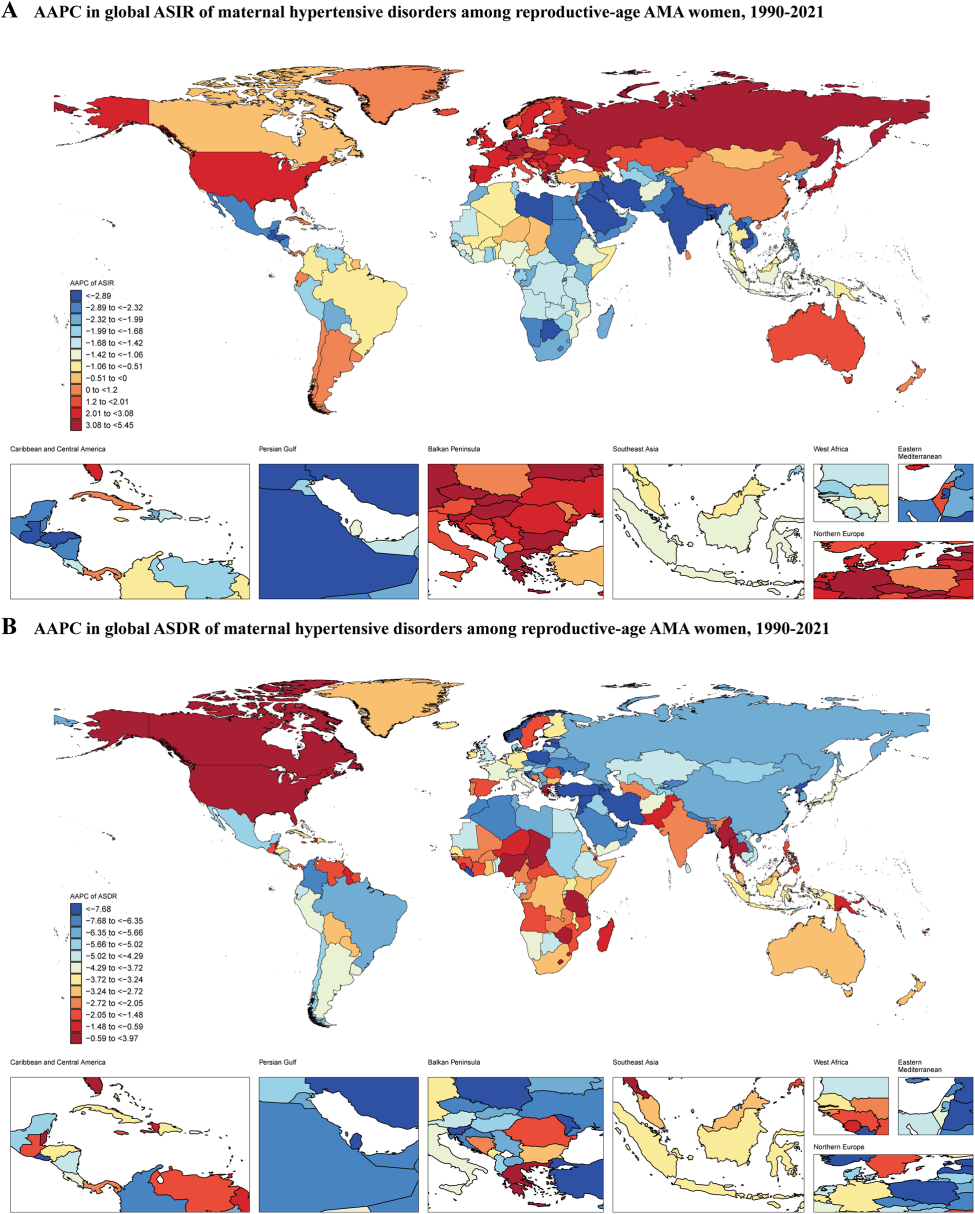
Average annual percentage change in global ASIR and ASDR of maternal hypertensive disorders among reproductive-age AMA women, 1990–2021. **(A)** ASIR; **(B)** ASDR; ASIR, age-standardized incidence rate; ASDR, age-standardized death rate; AMA, advanced maternal age.

The primary outcome measures were the age-standardized incidence rate (ASIR) and age-standardized death rate (ASDR). ASIR represents the number of new cases of maternal hypertensive disorders per 100,000 population, adjusted for differences in age structure across populations and time periods. ASDR represents the number of deaths from maternal hypertensive disorders per 100,000 population, similarly age-standardized to allow for valid comparisons across different populations and time periods. From 1990 to 2021, the global ASIR of MHD decreased from 568.10 (95% UI: 412.06–738.55) to 491.49 (95% UI: 368.78–619.84) per 100,000 population, with an average annual percentage change (AAPC) of −0.46% (95% CI: −0.54% to −0.38%) (Figure 1A, Table 1). During the same period, the ASDR declined from 2.57 (95% UI: 2.23–2.97) to 1.44 (95% UI: 1.19–1.76) per 100,000 population, with an AAPC of −1.83% (95% CI: −1.99% to −1.67%) (Figure 1B, Table 1).

**Table 1:** ASIR and ASDR of maternal hypertensive disorders among women of advanced maternal age in 1990 and 2021, and average annual percent change (1990–2021) at global and socio-demographic index regional levels.

Location	ASIR per 100,000 population			ASDR per 100,000 population		
	ASIR (95% uncertainty interval), 1990	ASIR (95% uncertainty interval), 2021	AAPC (95% CI), 1990–2021	ASDR (95% uncertainty interval), 1990	ASDR (95% uncertainty interval), 2021	AAPC (95% CI), 1990–2021
Global	568.10 (412.06 to 738.55)	491.49 (368.78 to 619.84)	−0.46 (−0.54 to −0.38)	2.57 (2.23 to 2.97)	1.44 (1.19 to 1.76)	−1.83 (−1.99 to −1.67)
High SDI	170.29 (118.88 to 236.90)	351.63 (257.26 to 464.17)	2.36 (2.24 to 2.47)	0.09 (0.07 to 0.11)	0.05 (0.04 to 0.06)	−1.87 (−2.35 to −1.38)
High-middle SDI	153.77 (101.10 to 217.97)	237.83 (171.05 to 317.23)	1.45 (1.32 to 1.59)	0.46 (0.35 to 0.58)	0.09 (0.07 to 0.11)	−5.13 (−5.5 to −4.76)
Middle SDI	422.58 (301.87 to 562.43)	312.22 (229.52 to 407.53)	−0.95 (−1.07 to −0.83)	1.50 (1.28 to 1.74)	0.62 (0.52 to 0.74)	−2.8 (−3.06 to −2.54)
Low-middle SDI	999.48 (715.06 to 1334.89)	508.41 (380.68 to 646.15)	−2.18 (−2.23 to −2.13)	5.40 (4.55 to 6.35)	2.38 (1.88 to 3.03)	−2.59 (−2.87 to −2.32)
Low SDI	2486.09 (1905.51 to 3019.84)	1605.80 (1240.79 to 1939.66)	−1.41 (−1.46 to −1.36)	13.44 (11.13 to 15.97)	6.14 (4.90 to 7.68)	−2.47 (−2.56 to −2.38)

ASIR, age-standardized incidence rate; ASDR, age-standardized death rate; AAPC, average annual percent change.

### Disparities across socio-demographic index regions

Significant disparities in disease burden were observed across Socio-demographic Index (SDI) regions. For ASIR, the low SDI region consistently showed the highest burden, though decreasing from 2,486.09 (95% UI: 1,905.51–3,019.84) per 100,000 population in 1990 to 1,605.80 (95% UI: 1,240.79–1,939.66) per 100,000 population in 2021, with an annual average percent change (AAPC) of −1.41% (95% CI: −1.46 to −1.36) (Table 1). In contrast, both high SDI and high-middle SDI regions showed increasing trends, with their ASIRs rising from 170.29 to 351.63 (AAPC: 2.36%, 95% CI: 2.24 to 2.47) and from 153.77 to 237.83 (AAPC: 1.45%, 95% CI: 1.32 to 1.59), respectively (Table 1). The middle SDI and low-middle SDI regions experienced decreasing trends, with AAPCs of −0.95% (95% CI: −1.07 to −0.83) and −2.18% (95% CI: −2.23 to −2.13), respectively (Table 1).

For ASDR, all SDI regions showed declining trends, but with varying magnitudes. The low SDI region maintained the highest ASDR, despite decreasing from 13.44 (95% UI: 11.13–15.97) per 100,000 population in 1990 to 6.14 (95% UI: 4.90–7.68) per 100,000 population in 2021 (AAPC: −2.47%, 95% CI: −2.56 to −2.38) (Table 1). The high-middle SDI region showed the most substantial decline (AAPC: −5.13%, 95% CI: −5.50 to −4.76), while the high SDI region maintained the lowest ASDR throughout the period, decreasing from 0.09 to 0.05 (AAPC: −1.87%, 95% CI: −2.35 to −1.38) (Table 1).

### Regional variations

At the Global Burden of Disease (GBD) regional level, Western Sub-Saharan Africa had the highest ASIR in 2021 at 2,234.25 (95% UI: 1,780.30–2,613.28) per 100,000 population, while East Asia had the lowest at 103.89 (95% UI: 69.45–150.93) (Supplementary Table S1). In 2021, the highest ASDR was observed in Central Sub-Saharan Africa at 8.86 (95% UI: 5.65–13.14) per 100,000 population, with Central Europe showing the lowest at 0.01 (95% UI: 0.01–0.02) (Supplementary Table S1). From 1990 to 2021, Eastern Europe experienced the fastest increase in ASIR with an AAPC of 3.17% (95% CI: 2.93% to 3.40%), while South Asia saw the most rapid decrease with an AAPC of −3.21% (95% CI: −3.40% to −3.03%) (Supplementary Table S1). East Asia demonstrated the most rapid decrease in ASDR with an AAPC of −6.35% (95% CI: −7.01% to −5.68%). High-income North America was the only region among the 21 GBD regions that showed an increase in ASDR, with an AAPC of 1.3% (95% CI: 0.49% to 2.12%) (Supplementary Table S1).

### National-level findings

At the national level, South Sudan had the highest ASIR in 2021 (3,404.51; 95% UI: 2,744.38–3,945.33), while Canada had the lowest (48.80; 95% UI: 32.15–75.37) (Supplementary Table S2, Supplementary Figure S1B). The Central African Republic had the highest ASDR (14.41; 95% UI: 7.74–24.58), whereas Slovenia had the lowest (0.002; 95% UI: 0.001–0.003) (Supplementary Table S2, SupplementaryFigure S1D). Between 1990 and 2021, Nepal showed the most substantial annual decline in ASIR (AAPC: −5.88%; 95% CI: −6.04% to −5.72%), while Czechia demonstrated the most substantial annual increase (AAPC: 5.45%; 95% CI: 5.12% to 5.79%) (Supplementary Table S2). Jordan exhibited the most substantial decrease in ASDR (AAPC: −11.34%; 95% CI: −11.72% to −10.96%), whereas Guam demonstrated the highest increase in ASDR (AAPC: 3.97%; 95% CI: 2.65% to 5.31%) over the study period (Supplementary Table S2).

### Correlation with socio-demographic development

**Figure 2:**
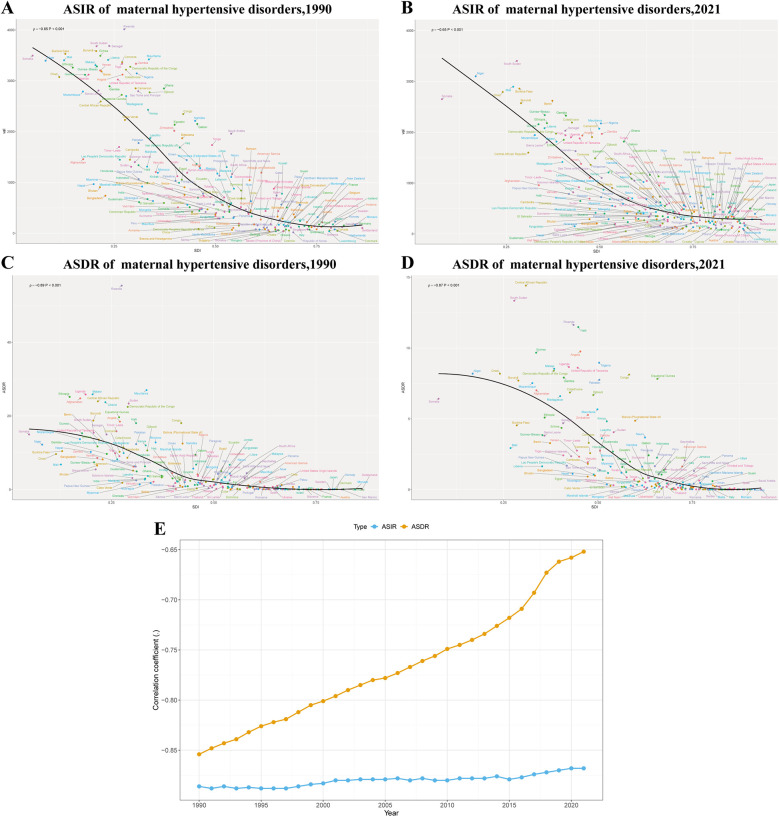
Correlation between SDI and ASIR and ASDR of maternal hypertensive disorders among reproductive-age AMA women at the national level in 1990 and 2021 and trends of correlation, 1990–2021. **(A)** The association between SDI and the ASIR, 1990; **(B)** The association between SDI and the ASIR, 2021; **(C)** The association between SDI and the ASDR,1990; **(D)** The association between SDI and the ASDR,2021. **(E)** Temporal trends in correlation coefficients between SDI and disease burden (ASIR and ASDR) from 1990 to 2021. SDI, socio-demographic Index; ASIR, age-standardized incidence rate; ASDR, age-standardized death rate; AMA, advanced maternal age.

A significant negative correlation was observed between MHD and socio-demographic development for both ASIR and ASDR across countries throughout the study period (1990–2021). For ASIR, the strength of the negative correlation between ASIR and SDI has gradually decreased over time, from *ρ* = −0.85 in 1990 to *ρ* = −0.65 in 2021 (*P* < 0.001) (Figures 2A,B, E), indicating that while countries with lower SDI generally exhibited higher incidence rates, this relationship has become relatively less pronounced over the past three decades. The correlation between ASDR and SDI has remained consistently strong throughout the study period (ranging from *ρ* = −0.89 in 1990 to *ρ* = −0.87 in 2021, *P* < 0.001) (Figures 2C,D and E), suggesting that ASDR decreased more consistently with increasing socio-demographic development.The temporal trend analysis further revealed that the correlation coefficient for ASIR steadily weakened over time, while that for ASDR remained relatively stable throughout the study period (Figure 2E), highlighting the divergent patterns in the relationship between socio-demographic development and disease incidence vs. mortality.

### Trends across SDI regions

**Figure 3:**
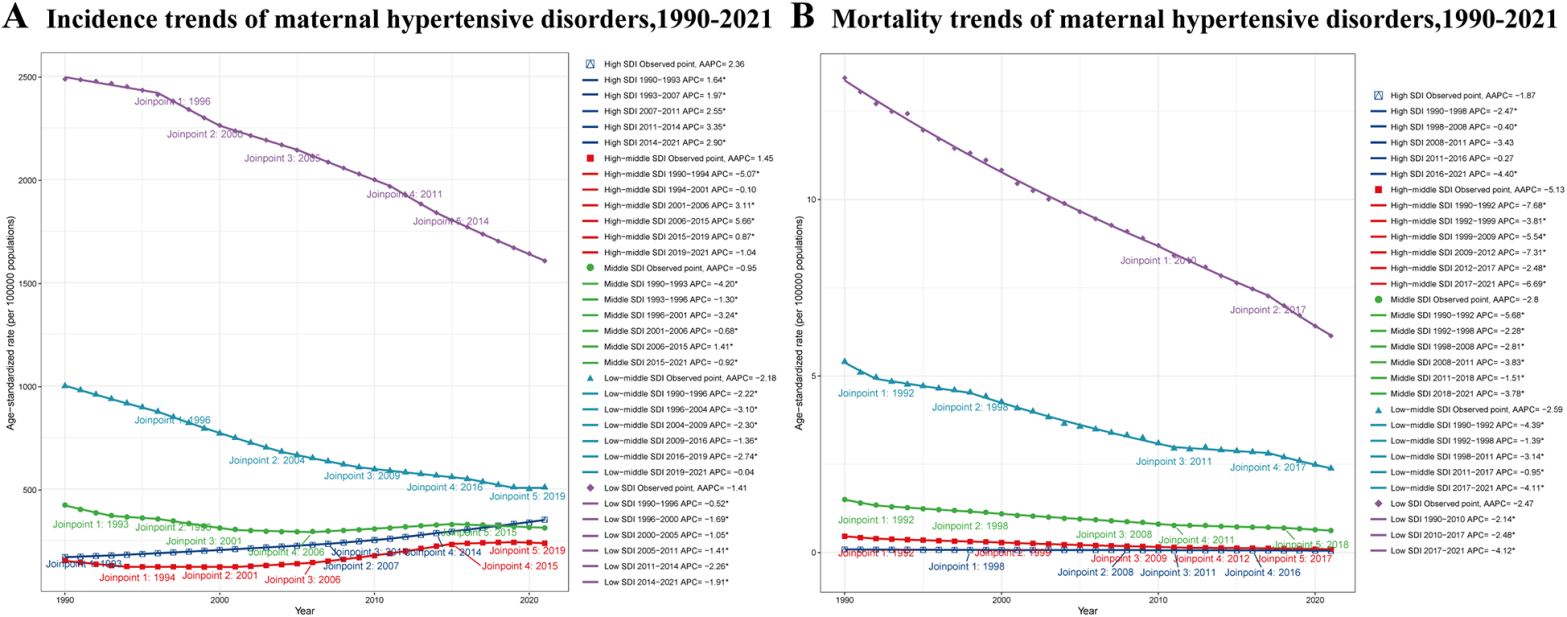
ASIR and ASDR trends of maternal hypertensive disorders among reproductive-age AMA women across different SDI regions, 1990–2021. **(A)** ASIR trends of maternal hypertensive disorders **(B)** ASDR trends of maternal hypertensive disorders. ASIR, age-standardized incidence rate; ASDR, age-standardized death rate; AMA, advanced maternal age; SDI, socio-demographic Index.

From 1990 to 2021, trends in ASIR varied across different SDI regions (Figure 3A). High SDI regions showed a consistent increase (AAPC: 2.36%). The trend accelerated from 1990 to 2014, peaking with an annual percent change (APC) of 3.35% during 2011–2014, before slightly decelerating to 2.90% from 2014 to 2021. High-middle SDI regions experienced overall growth (AAPC: 1.45%) but with fluctuations. After an initial decline (APC: −5.07%) from 1990 to 1994, the trend reversed, reaching a 5.66% annual increase from 2006 to 2015, before stabilizing and slightly declining in recent years. Middle SDI regions showed a slight overall decrease (AAPC: −0.95%), characterized by periods of decline from 1990 to 2001, followed by an increase (APC: 1.41%) from 2006 to 2015, and then declining again. Low-middle SDI regions demonstrated a consistent decrease (AAPC: −2.18%), with the most rapid decline (APC: −3.10%) between 1996 and 2004. Low SDI regions also showed an overall decrease (AAPC: −1.41%), with varying rates over time and the most rapid decrease (APC: −2.26%) observed from 2011 to 2014.

The ASDR exhibited a decreasing trend across all SDI regions from 1990 to 2021 (Figure 3B). High SDI regions experienced the smallest decline (AAPC: −1.87%), with periods of slower decrease (APC: −0.40% from 1998 to 2008) and more rapid decline (APC: −4.40% from 2016 to 2021). High-middle SDI regions showed the most substantial decrease (AAPC: −5.13%), with rapid declines (APC: −7.68%) from 1990 to 1992 and (APC: −6.69%) from 2017 to 2021. Middle SDI regions demonstrated a consistent decline (AAPC: −2.80%), with the most rapid decrease (APC: −5.68%) observed from 1990 to 1992. Low-middle SDI regions showed a steady decrease (AAPC: −2.59%), with rapid declines from 1990 to 1992 (APC: −4.39%) and from 2017 to 2021 (APC: −4.11%). Low SDI regions experienced a consistent decline (AAPC: −2.47%), with the rate of decrease accelerating in recent years (APC: −4.12% from 2017 to 2021).

### Health inequalities among reproductive-age AMA women

**Figure 4:**
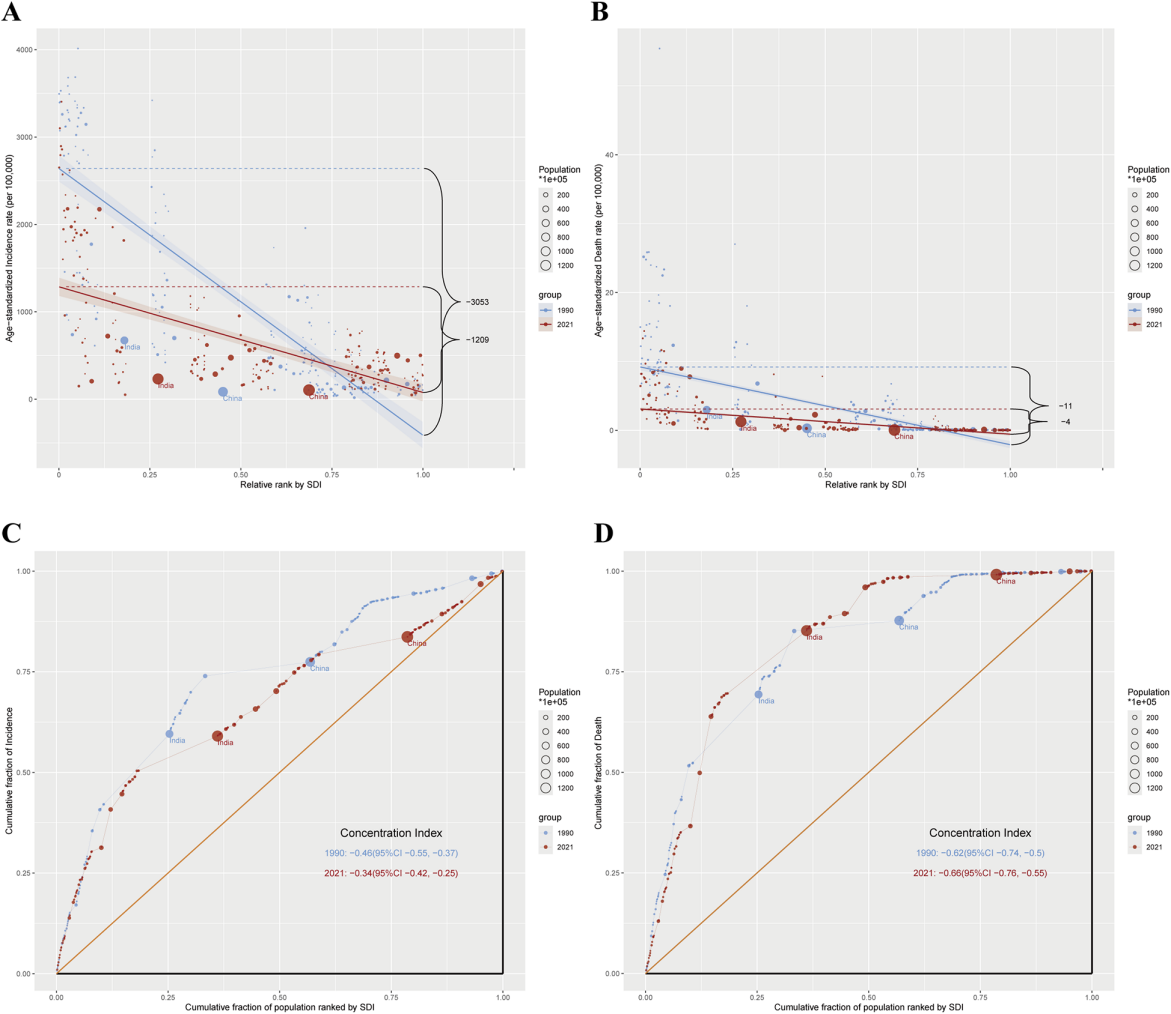
Absolute and relative cross-country inequality in ASIR and ASDR of maternal hypertensive disorders among reproductive-age AMA women, 1990–2021. **(A)** Health inequality regression curves for ASIR of maternal hypertensive disorders among reproductive-age AMA women; **(B)** Health inequality regression curves for ASDR of maternal hypertensive disorders among reproductive-age AMA women; **(C)** Concentration curves for ASIR of maternal hypertensive disorders among reproductive-age AMA women; **(D)** Concentration curves for ASDR of maternal hypertensive disorders among reproductive-age AMA women; ASIR, age-standardized incidence rate; ASDR, age-standardized death rate; AMA, advanced maternal age.

Significant inequalities in the burden of MHD among reproductive-age women of advanced maternal age were observed across different SDI levels from 1990 to 2021. The ASIR showed a consistent decrease, with notable disparities between countries at different SDI levels. In 1990, the SII for ASIR was −3,052.73 (95% CI: −3,329.55 to −2,775.91) per 100,000 women, indicating substantial absolute inequality between countries with the lowest and highest SDI (Figure 4A). By 2021, the SII had reduced to −1,209.36 (95% CI: −1,393.12 to −1,025.61) per 100,000 women, representing a 60.4% reduction (Figure 4A).

The SII for ASDR showed a similar trend. In 1990, the SII was −11.29 deaths per 100,000 women (95% CI: −12.38 to −10.20), indicating that countries with the lowest SDI experienced approximately 11 more deaths per 100,000 women compared to those with the highest SDI (Figure 4B). By 2021, the SII had decreased to −3.66 deaths per 100,000 women (95% CI: −4.13 to −3.20) (Figure 4B). Although inequality persisted, it decreased over time. The consistently negative SII values indicate that the burden among reproductive-age AMA women was consistently higher in countries with lower SDI levels.

The concentration index for ASIR among reproductive-age AMA women indicated a disproportionate burden among poorer populations, with slight improvement over time. The global concentration index for incidence was −0.46 (95% CI: −0.55 to −0.37) in 1990, improving to −0.34 (95% CI: −0.42 to −0.25) in 2021 (Figure 4C). For ASDR, the concentration index showed a marginal increase in inequality, from −0.62 (95% CI: −0.74 to −0.50) in 1990 to −0.66 (95% CI: −0.76 to −0.55) in 2021 (Figure 4D).

## Discussion

The main objective of this study was to analyze temporal trends, disparities, and health inequalities in the burden of MHD among reproductive-age women of AMA between 1990 and 2021. Our findings revealed a global decline in both the ASIR and ASDR of MHD over the study period. Specifically, the ASIR decreased from 568.10 to 491.49 per 100,000 population, and the ASDR decreased from 2.57 to 1.44 per 100,000 population, with annual percentage changes (AAPC) of −0.46% and −1.83%, respectively. Despite this overall decline, significant disparities in incidence and mortality persisted across Socio-demographic Index (SDI) regions. Notably, low SDI regions consistently had higher ASIR and ASDR compared to other regions, while high and high-middle SDI regions demonstrated increasing trends in ASIR. Although absolute inequalities in both incidence and mortality have decreased over time, relative inequalities in mortality have persisted or even increased.

The observed overall decline in global ASIR and ASDR of MHD may be attributed to improvements in healthcare access, increased awareness of maternal health risks, and advancements in obstetric care (7, 27, 28). The significant disparities across SDI regions reflect underlying socioeconomic, healthcare, and policy differences. Low SDI regions bore the highest burden of MHD, likely due to limited access to quality maternal healthcare, inadequate healthcare infrastructure, and socioeconomic challenges. This high burden underscores the need for strengthened maternal healthcare services and policies targeting maternal health risks for reproductive-age AMA women in these regions (29). However, contrasting trends were observed across different SDI regions. The increasing ASIR trends in high and high-middle SDI regions also warrant attention. Factors such as rising maternal age, increased prevalence of obesity, and associated comorbidities like diabetes and cardiovascular disease are likely contributing to this trend (14, 30, 31). Studies from developed countries have reported an increased risk of hypertensive disorders in pregnancies among older mothers, highlighting the need for preventive strategies focused on these risk factors (32–36). These risk factors, particularly obesity and its associated comorbidities, are not limited to developed nations but are increasing globally, affecting both high and low SDI regions (37). However, the impact of these conditions on MHD trends appears to be offset by different factors across regions. Particularly in lower SDI regions, despite the rising prevalence of obesity and resource constraints, the aforementioned improvements in healthcare infrastructure, enhanced access to prenatal care services, better awareness and management of traditional risk factors, coupled with targeted maternal health programs and early identification of high-risk pregnancies, have likely contributed to their declining ASIR trends (38).

Our findings indicate significant health inequalities in the burden of MHD among reproductive-age AMA women, with consistent disparities observed across countries of different SDI levels. Although absolute inequalities in both incidence and mortality have decreased over time, countries with lower SDI levels continue to bear a disproportionately higher burden. The concentration index for ASIR improved from 1990 to 2021, indicating a reduction in relative inequalities in incidence. However, for ASDR, the concentration index slightly worsened during the same period, suggesting that relative inequalities in mortality have persisted or increased. This contrasting trend between ASIR and ASDR highlights the need for continued efforts to address health disparities, particularly in mortality outcomes. To effectively address these inequalities, efforts should focus on improving healthcare access in low SDI regions, ensuring equitable distribution of resources, and addressing underlying socioeconomic determinants that contribute to health disparities.

The strengths of this study include the use of comprehensive global data from the GBD 2021 study and the analysis of long-term trends in MHD among reproductive-age AMA women. However, limitations exist, such as potential variations in data quality, underreporting in some regions, and reliance on modeled estimates, which may affect the accuracy of the findings. These limitations should be considered when interpreting the results, and future studies should aim to improve data quality and coverage.

The findings of this study have important implications for global maternal health policies. Targeted interventions are needed to address the rising burden of MHD in high and high-middle SDI regions and to reduce health inequalities in low SDI regions. Policymakers should focus on improving healthcare access, promoting preventive measures, and addressing socioeconomic determinants of health to enhance maternal outcomes among reproductive-age AMA women.

## Conclusion

This study highlights the global burden, inequalities, and trends of MHD among reproductive-age AMA women from 1990 to 2021. While progress has been made in reducing the overall burden of MHD, significant disparities persist, particularly in low SDI regions. Continued efforts are essential to address these inequalities and improve maternal health outcomes for reproductive-age AMA women worldwide.

## Data Availability

The original contributions presented in the study are included in the article/Supplementary Material, further inquiries can be directed to the corresponding author.
