# Supplementary material for: Global burden, trends and inequalities of maternal hypertensive disorders among reproductive-age women of advanced maternal age, 1990–2021: a population-based study
Source: Front Glob Womens Health. 2025 Mar 6;6:1513909. doi: 10.3389/fgwh.2025.1513909 (PMC11922729; doi:10.3389/fgwh.2025.1513909)
Supplement: Supplementary file 1 [file Datasheet1.pdf]

Supplementary appendix

Supplement to: Global burden, trends and inequalities of maternal hypertensive disorders among women of advanced maternal age, 1990-2021: A population-based study

Xuanyu Zhao[a], Weimin Kong[b]*, Yan Jiang[a], Feng Sui[a]

[a] Department of Maternal Intensive Care Unit, Beijing Obstetrics and Gynecology Hospital, Capital Medical University; Beijing Maternal and Child Health Care Hospital, Beijing 100006, China.

[b] Department of Gynecology, Beijing Obstetrics and Gynecology Hospital, Capital Medical University; Beijing Maternal and Child Health Care Hospital, Beijing 100026, China.

*Corresponding author

E-mail addresses: kwm1967@ccmu.edu.cn

Supplementary file

Supplementary Table 1. ASIR and ASDR of maternal hypertensive disorders among women of advanced maternal age in 1990 and 2021, and average annual percent change (1990-2021) at GBD regional level. 1

Supplementary Table 2. ASIR and ASDR of maternal hypertensive disorders among women of advanced maternal age in 1990 and 2021, and average annual percent change (1990-2021) at national level. 3

Supplementary Fig 1. ASIR and ASDR of maternal hypertensive disorders among women of AMA across all countries in 1990 and 2021. 18

Supplementary Table 3. Supplementary table 3: Regional Hierarchy and Country Classification in the GBD 2021 Study 19

Supplementary table 1: ASIR and ASDR of maternal hypertensive disorders among women of advanced maternal age in 1990 and 2021, and average annual percent change (1990-2021) at GBD regional level.

location	1990ASIR	2021ASIR	AAPC of ASIR,1990-2021 (95%CI)	1990ASDR	2021ASDR	AAPC of ASDR,1990-2021 (95%CI)
High-income Asia Pacific	134.42(97.99 to 176.43)	317.44(246.95 to 397.86)	2.79 (2.7 to 2.89)	0.06(0.05 to 0.08)	0.01(0.01 to 0.02)	-4.61 (-5.51 to -3.71)
Central Asia	296.45(192.05 to 432.99)	264.48(180.39 to 360.56)	-0.37 (-0.49 to -0.24)	0.99(0.83 to 1.17)	0.21(0.17 to 0.25)	-4.82 (-5.52 to -4.11)
East Asia	84.63(55.64 to 126.73)	103.89(69.45 to 150.93)	0.69 (0.37 to 1)	0.32(0.21 to 0.44)	0.04(0.03 to 0.06)	-6.35 (-7.01 to -5.68)
Southeast Asia	706.48(478.10 to 972.30)	452.07(319.66 to 611.23)	-1.43 (-1.47 to -1.4)	3.58(2.71 to 4.67)	1.40(1.07 to 1.81)	-2.94 (-3.12 to -2.77)
Oceania	967.91(640.47 to 1397.63)	765.92(514.84 to 1090.07)	-0.76 (-0.79 to -0.73)	2.18(1.17 to 3.55)	1.63(1.00 to 2.56)	-0.87 (-1.26 to -0.49)
South Asia	779.28(535.54 to 1081.99)	283.93(196.48 to 385.86)	-3.21 (-3.4 to -3.03)	4.32(3.44 to 5.29)	1.95(1.40 to 2.67)	-2.51 (-2.89 to -2.14)
Caribbean	464.92(310.01 to 654.23)	374.54(256.58 to 522.03)	-0.69 (-0.76 to -0.61)	3.66(2.49 to 5.16)	3.54(2.07 to 5.61)	-0.04 (-0.33 to 0.25)
Andean Latin America	437.71(341.65 to 566.61)	352.36(306.78 to 416.34)	-0.69 (-0.78 to -0.6)	5.84(4.47 to 7.27)	1.75(1.21 to 2.48)	-3.84 (-4.39 to -3.29)
Central Latin America	863.83(672.50 to 1083.27)	444.92(371.61 to 530.44)	-2.11 (-2.15 to -2.06)	1.87(1.61 to 2.14)	0.51(0.40 to 0.65)	-3.97 (-5.1 to -2.83)
Tropical Latin America	382.19(268.09 to 526.97)	292.61(218.24 to 380.36)	-0.85 (-0.93 to -0.76)	2.77(2.22 to 3.39)	0.39(0.30 to 0.48)	-6.03 (-6.58 to -5.48)
Southern Latin America	438.72(279.66 to 637.85)	518.34(396.93 to 666.14)	0.53 (0.46 to 0.61)	0.65(0.51 to 0.82)	0.15(0.11 to 0.20)	-4.48 (-5.58 to -3.37)

North Africa and Middle East	1172.46(791.52 to 1617.94)	563.52(375.86 to 777.16)	-2.34 (-2.39 to -2.29)	5.64(4.37 to 6.96)	0.82(0.58 to 1.13)	-6.05 (-6.21 to -5.88)
Central Sub-Saharan Africa	3148.27(2395.72 to 3835.60)	1884.78(1394.48 to 2369.43)	-1.65 (-1.68 to -1.62)	21.35(13.66 to 31.21)	8.86(5.65 to 13.14)	-2.73 (-2.94 to -2.53)
Eastern Sub-Saharan Africa	3124.78(2436.82 to 3695.92)	1926.65(1514.52 to 2265.91)	-1.54 (-1.58 to -1.5)	19.00(15.35 to 22.96)	6.77(5.29 to 8.53)	-3.25 (-3.44 to -3.06)
Southern Sub-Saharan Africa	1381.01(1027.85 to 1682.11)	692.16(525.77 to 841.29)	-2.21 (-2.27 to -2.16)	3.87(2.74 to 5.34)	2.06(1.47 to 2.79)	-2.1 (-3.21 to -0.97)
Western Sub-Saharan Africa	3170.05(2491.75 to 3712.33)	2234.25(1780.30 to 2613.28)	-1.14 (-1.25 to -1.03)	8.29(6.40 to 10.40)	6.58(4.71 to 9.03)	-0.72 (-0.89 to -0.54)
Central Europe	84.85(53.75 to 126.72)	159.88(111.18 to 220.25)	2.09 (1.85 to 2.34)	0.07(0.06 to 0.08)	0.01(0.01 to 0.02)	-5.04 (-6.81 to -3.24)
Eastern Europe	127.78(82.31 to 183.76)	329.95(222.15 to 452.11)	3.17 (2.93 to 3.4)	0.13(0.11 to 0.16)	0.02(0.01 to 0.02)	-6.31 (-6.92 to -5.71)
Western Europe	134.23(89.62 to 193.80)	329.75(232.97 to 450.40)	2.95 (2.91 to 2.98)	0.05(0.05 to 0.06)	0.02(0.01 to 0.02)	-3.95 (-4.7 to -3.18)
High-income North America	200.42(140.70 to 281.62)	451.30(351.68 to 569.96)	2.63 (2.55 to 2.7)	0.06(0.04 to 0.08)	0.08(0.06 to 0.12)	1.3 (0.49 to 2.12)
Australasia	234.69(174.52 to 305.52)	347.19(229.95 to 506.94)	1.28 (1.08 to 1.49)	0.03(0.03 to 0.05)	0.01(0.01 to 0.02)	-3.12 (-4.83 to -1.38)

ASIR, age-standardized incidence rate. ASDR, age-standardized death rate. AAPC, average annual percent change. GBD, Global Burden of Disease

Supplementary table 2: ASIR and ASDR of maternal hypertensive disorders among women of advanced maternal age in 1990 and 2021, and average annual percent change (1990-2021) at national level.

location	1990ASIR	2021ASIR	AAPC of ASIR,1990-2021(95%CI)	1990ASDR	2021ASDR	AAPC of ASDR,1990-2021(95%CI)
Afghanistan	1457.16(935.88 to 2177.70)	958.64(608.71 to 1437.35)	-1.35(-1.39 to -1.3)	24.78(13.98 to 40.08)	7.01(3.66 to 12.17)	-4.02(-4.17 to -3.86)
Albania	191.16(119.71 to 287.11)	120.29(74.82 to 184.69)	-1.45(-1.59 to -1.3)	0.06(0.04 to 0.09)	0.01(0.01 to 0.02)	-5.08(-5.8 to -4.36)
Algeria	1312.70(828.84 to 1926.53)	952.87(597.26 to 1395.19)	-1.02(-1.08 to -0.96)	4.11(2.47 to 6.38)	0.48(0.27 to 0.77)	-6.65(-6.92 to -6.38)
American Samoa	1192.28(833.64 to 1577.00)	802.31(586.97 to 1014.81)	-1.28(-1.32 to -1.23)	2.11(1.13 to 3.42)	1.63(0.92 to 2.76)	-0.82(-1.68 to 0.05)
Andorra	125.76(79.44 to 192.95)	240.57(147.46 to 360.85)	2.12(2.06 to 2.18)	0.03(0.01 to 0.06)	0.01(0.00 to 0.02)	-4.26(-4.67 to -3.84)
Angola	3121.43(2303.04 to 3888.95)	1934.62(1408.06 to 2438.96)	-1.54(-1.59 to -1.49)	18.46(10.17 to 30.95)	9.76(5.47 to 16.58)	-1.97(-2.35 to -1.6)
Antigua and Barbuda	269.52(180.78 to 390.29)	247.96(167.34 to 349.47)	-0.27(-0.36 to -0.18)	0.34(0.25 to 0.45)	0.15(0.10 to 0.19)	-2.46(-4.25 to -0.65)
Argentina	450.93(282.67 to 664.26)	487.44(321.23 to 692.52)	0.27(0.21 to 0.34)	0.70(0.50 to 0.96)	0.19(0.13 to 0.26)	-4.14(-5.65 to -2.61)
Armenia	104.83(67.18 to 157.86)	141.41(90.10 to 209.40)	0.97(0.85 to 1.09)	0.34(0.25 to 0.46)	0.02(0.01 to 0.03)	-8.79(-10.98 to -6.54)
Australia	219.00(165.25 to 285.84)	329.85(206.01 to 499.25)	1.28(0.87 to 1.69)	0.04(0.03 to 0.05)	0.01(0.01 to 0.02)	-2.88(-3.98 to -1.76)
Austria	76.56(73.13 to 80.08)	117.05(108.80 to 125.07)	1.37(1.31 to 1.43)	0.04(0.03 to 0.05)	0.01(0.01 to 0.01)	-4.61(-5.49 to -3.73)

Azerbaijan	172.94(107.79 to 259.38)	112.77(72.75 to 164.31)	-1.4(-1.55 to -1.25)	0.52(0.35 to 0.72)	0.04(0.02 to 0.07)	-7.67(-8.29 to -7.04)
Bahamas	370.09(254.26 to 513.51)	340.30(234.91 to 470.61)	-0.26(-0.38 to -0.14)	0.71(0.53 to 0.94)	0.30(0.20 to 0.43)	-2.88(-3.73 to -2.01)
Bahrain	1044.32(669.31 to 1463.37)	434.58(287.50 to 608.80)	-2.82(-2.94 to -2.7)	1.14(0.68 to 1.73)	0.15(0.08 to 0.25)	-6.48(-7.44 to -5.52)
Bangladesh	738.92(498.90 to 1057.31)	205.87(138.46 to 290.20)	-4.02(-4.17 to -3.88)	8.19(5.73 to 11.30)	0.99(0.55 to 1.60)	-6.44(-7.19 to -5.68)
Barbados	329.40(226.82 to 452.64)	317.41(219.60 to 435.99)	-0.08(-0.19 to 0.03)	0.30(0.22 to 0.39)	0.16(0.11 to 0.23)	-1.76(-4.22 to 0.77)
Belarus	81.30(50.27 to 123.75)	239.86(151.39 to 357.92)	3.6(3.44 to 3.75)	0.12(0.09 to 0.15)	0.02(0.01 to 0.03)	-5.9(-7.51 to -4.26)
Belgium	119.05(75.88 to 177.62)	343.52(215.99 to 515.95)	3.46(3.28 to 3.64)	0.04(0.03 to 0.05)	0.01(0.01 to 0.02)	-3.31(-3.61 to -3.02)
Belize	904.52(591.96 to 1313.83)	398.14(279.53 to 536.63)	-2.6(-2.66 to -2.53)	0.72(0.53 to 0.93)	0.59(0.43 to 0.79)	-0.49(-3.95 to 3.1)
Benin	3185.22(2519.16 to 3717.75)	2620.72(2137.40 to 3038.71)	-0.62(-0.65 to -0.59)	13.56(8.73 to 19.40)	3.28(1.89 to 5.08)	-4.49(-4.72 to -4.27)
Bermuda	317.67(222.33 to 431.08)	567.75(394.49 to 760.46)	1.91(1.84 to 1.98)	0.11(0.08 to 0.15)	0.01(0.01 to 0.01)	-7.83(-9.35 to -6.29)
Bhutan	906.26(589.90 to 1302.04)	290.39(192.29 to 415.09)	-3.63(-3.74 to -3.52)	6.53(3.69 to 10.57)	0.92(0.47 to 1.58)	-6.11(-6.26 to -5.97)
Bolivia (Plurinational State of)	756.09(500.85 to 1110.72)	393.48(266.80 to 577.29)	-2.07(-2.16 to -1.98)	12.51(8.21 to 18.36)	4.87(2.73 to 7.96)	-2.98(-3.46 to -2.5)
Bosnia and Herzegovina	67.44(42.79 to 100.98)	111.24(70.01 to 172.57)	1.61(1.53 to 1.7)	0.03(0.02 to 0.04)	0.01(0.01 to 0.02)	-2.4(-3.26 to -1.54)
Botswana	1870.69(1354.68 to 2372.11)	727.28(551.56 to 883.71)	-3(-3.06 to -2.95)	1.03(0.49 to 1.87)	0.23(0.11 to 0.42)	-4.76(-6.78 to -2.7)

Brazil	373.20(261.88 to 513.24)	286.93(214.94 to 371.44)	-0.83(-0.92 to -0.75)	2.78(2.21 to 3.41)	0.37(0.28 to 0.47)	-6.13(-6.71 to -5.56)
Brunei Darussalam	289.05(194.72 to 424.56)	233.47(159.10 to 342.26)	-0.67(-0.84 to -0.5)	1.26(0.72 to 2.03)	0.23(0.13 to 0.37)	-5.46(-5.73 to -5.18)
Bulgaria	33.59(23.35 to 48.83)	116.72(70.85 to 180.11)	4.09(3.36 to 4.83)	0.10(0.07 to 0.13)	0.03(0.02 to 0.05)	-3.05(-7.2 to 1.28)
Burkina Faso	3529.90(2756.76 to 4194.28)	2858.78(2239.02 to 3395.64)	-0.69(-0.73 to -0.65)	10.44(6.12 to 15.78)	4.54(2.48 to 7.49)	-2.56(-3.24 to -1.87)
Burundi	3584.63(2806.26 to 4253.46)	2572.13(1938.65 to 3133.16)	-1.06(-1.08 to -1.05)	18.75(10.57 to 29.44)	7.70(4.39 to 12.27)	-2.81(-3.1 to -2.53)
Cabo Verde	2229.52(1762.11 to 2612.10)	904.60(741.31 to 1037.74)	-2.89(-2.98 to -2.8)	1.84(1.04 to 3.00)	0.27(0.14 to 0.48)	-5.94(-6.28 to -5.59)
Cambodia	1447.16(975.39 to 2050.71)	492.75(325.99 to 712.99)	-3.42(-3.48 to -3.35)	8.64(5.18 to 13.49)	1.82(0.95 to 3.12)	-4.87(-5.16 to -4.57)
Cameroon	2850.91(2312.20 to 3248.79)	1905.49(1569.71 to 2164.74)	-1.29(-1.34 to -1.25)	5.14(3.25 to 7.69)	2.57(1.39 to 4.58)	-2.22(-2.45 to -2)
Canada	56.79(42.02 to 77.18)	48.80(32.15 to 75.37)	-0.42(-1.51 to 0.69)	0.02(0.01 to 0.03)	0.02(0.02 to 0.03)	0.99(-0.22 to 2.23)
Central African Republic	2592.66(1925.12 to 3196.51)	1600.62(1155.87 to 2065.50)	-1.55(-1.57 to -1.53)	23.98(14.87 to 34.98)	14.41(7.74 to 24.58)	-1.62(-1.78 to -1.47)
Chad	3073.46(2387.69 to 3660.35)	2793.54(2145.90 to 3401.35)	-0.31(-0.35 to -0.28)	9.65(6.04 to 14.31)	8.19(4.76 to 12.78)	-0.49(-0.82 to -0.15)
Chile	421.62(269.74 to 611.38)	606.46(594.50 to 620.37)	1.2(1.13 to 1.26)	0.59(0.45 to 0.77)	0.08(0.06 to 0.11)	-5.65(-6.14 to -5.16)
China	85.15(56.01 to 127.42)	104.35(69.86 to 151.53)	0.73(0.41 to 1.06)	0.29(0.19 to 0.39)	0.04(0.03 to 0.05)	-6.23(-6.91 to -5.55)

Colombia	470.30(338.89 to 650.67)	348.82(256.58 to 482.03)	-0.92(-1.09 to -0.75)	2.03(1.51 to 2.68)	0.27(0.18 to 0.38)	-6.69(-9.26 to -4.04)
Comoros	3367.21(2675.13 to 3914.28)	1359.38(1088.78 to 1575.68)	-2.89(-2.93 to -2.86)	10.26(5.12 to 17.21)	3.30(1.77 to 5.68)	-3.64(-4.77 to -2.5)
Congo	2345.08(1779.08 to 2836.82)	1180.15(909.30 to 1427.58)	-2.2(-2.27 to -2.12)	18.05(10.16 to 28.55)	8.13(4.18 to 14.67)	-2.47(-2.87 to -2.08)
Cook Islands	686.38(449.65 to 969.80)	500.68(363.22 to 640.50)	-1.02(-1.1 to -0.93)	0.04(0.02 to 0.08)	0.01(0.01 to 0.02)	-4.01(-4.31 to -3.7)
Costa Rica	524.21(369.30 to 733.59)	316.71(232.14 to 417.82)	-1.58(-1.73 to -1.43)	0.40(0.29 to 0.52)	0.10(0.07 to 0.14)	-4.21(-4.8 to -3.62)
Coted'Ivoire	3218.33(2537.29 to 3790.72)	2192.20(1720.70 to 2565.11)	-1.23(-1.26 to -1.21)	11.46(7.19 to 17.93)	6.86(3.91 to 11.60)	-1.61(-1.89 to -1.33)
Croatia	45.24(30.08 to 68.31)	103.49(89.93 to 117.95)	2.7(2.54 to 2.85)	0.07(0.05 to 0.09)	0.01(0.00 to 0.01)	-7.27(-9.23 to -5.26)
Cuba	150.67(104.55 to 207.59)	204.11(139.86 to 280.78)	1(0.8 to 1.2)	0.23(0.17 to 0.29)	0.07(0.05 to 0.09)	-3.7(-4.89 to -2.5)
Cyprus	77.87(51.45 to 113.80)	154.65(98.27 to 234.04)	2.14(1.59 to 2.7)	0.11(0.06 to 0.19)	0.01(0.01 to 0.02)	-6.8(-7.15 to -6.44)
Czechia	55.12(36.18 to 80.08)	280.69(174.91 to 421.29)	5.45(5.12 to 5.79)	0.04(0.03 to 0.05)	0.01(0.00 to 0.01)	-6.37(-7 to -5.73)
Democratic People's Republic of Korea	106.32(65.68 to 162.54)	51.33(33.75 to 77.75)	-2.29(-2.36 to -2.22)	2.70(1.39 to 4.52)	0.21(0.11 to 0.38)	-7.92(-8.12 to -7.73)
Democratic Republic of the Congo	3276.47(2484.63 to 3997.97)	1973.84(1427.64 to 2513.93)	-1.62(-1.67 to -1.58)	22.47(13.09 to 35.10)	8.40(4.73 to 13.55)	-3.09(-3.38 to -2.79)
Denmark	47.42(32.70 to 67.15)	119.19(78.22 to 172.43)	3.07(2.71 to 3.43)	0.08(0.05 to 0.11)	0.02(0.01 to 0.02)	-5.26(-7.99 to -2.45)
Djibouti	2772.76(2169.45 to 3307.56)	1487.84(1181.99 to 1754.13)	-2(-2.04 to -1.96)	10.63(6.00 to 17.31)	6.68(3.49 to 11.62)	-1.47(-1.56 to -1.38)

Dominica	840.42(566.38 to 1184.23)	401.29(281.11 to 543.57)	-2.35(-2.48 to -2.23)	0.06(0.04 to 0.10)	0.05(0.03 to 0.09)	-0.67(-1.61 to 0.27)
Dominican Republic	421.18(282.04 to 597.36)	268.50(186.45 to 373.63)	-1.43(-1.48 to -1.38)	2.39(1.53 to 3.38)	0.79(0.45 to 1.28)	-3.58(-4.98 to -2.17)
Ecuador	553.83(522.25 to 587.34)	734.22(719.34 to 749.38)	0.92(0.86 to 0.97)	5.00(3.81 to 6.35)	1.08(0.70 to 1.57)	-4.55(-7.22 to -1.82)
Egypt	1053.29(713.96 to 1483.71)	452.34(309.59 to 622.48)	-2.68(-2.73 to -2.63)	2.68(1.68 to 3.88)	0.55(0.31 to 0.87)	-4.95(-5.49 to -4.41)
El Salvador	595.70(421.15 to 833.34)	310.91(225.58 to 422.56)	-2.09(-2.34 to -1.84)	3.30(2.09 to 4.87)	0.20(0.11 to 0.33)	-8.55(-10.06 to -7.01)
Equatorial Guinea	2723.72(1997.52 to 3385.04)	1132.82(928.83 to 1288.02)	-2.81(-2.88 to -2.74)	19.64(9.47 to 34.38)	7.82(3.71 to 14.50)	-2.82(-3.38 to -2.26)
Eritrea	3491.78(2690.75 to 4177.33)	1852.49(1398.17 to 2241.88)	-2.04(-2.07 to -2)	11.71(6.94 to 18.27)	4.36(2.11 to 7.98)	-3.07(-3.43 to -2.72)
Estonia	138.74(87.04 to 207.78)	514.91(335.95 to 734.83)	4.38(3.89 to 4.87)	0.07(0.05 to 0.09)	0.00(0.00 to 0.01)	-8.33(-9.65 to -6.99)
Eswatini	2134.28(1630.84 to 2550.14)	1087.72(843.10 to 1294.28)	-2.15(-2.17 to -2.14)	3.02(1.60 to 5.11)	1.98(0.86 to 3.72)	-1.35(-1.83 to -0.86)
Ethiopia	3260.19(2507.44 to 3989.24)	2178.97(1728.67 to 2597.96)	-1.27(-1.38 to -1.15)	25.18(17.65 to 34.80)	5.09(3.26 to 7.46)	-4.88(-5.14 to -4.63)
Fiji	419.96(277.09 to 605.90)	549.69(371.55 to 757.34)	0.86(0.79 to 0.93)	1.64(0.89 to 2.80)	1.39(0.73 to 2.39)	-0.54(-1.4 to 0.32)
Finland	172.23(108.73 to 258.93)	302.53(189.00 to 461.54)	1.86(1.72 to 2)	0.03(0.02 to 0.04)	0.01(0.01 to 0.01)	-3.36(-5.34 to -1.33)
France	129.87(81.42 to 195.18)	329.52(202.30 to 507.28)	3.08(3.03 to 3.13)	0.06(0.05 to 0.08)	0.02(0.01 to 0.02)	-3.76(-4.39 to -3.12)
Gabon	2150.77(1619.34 to 2597.18)	1163.00(928.59 to 1358.69)	-1.96(-2.03 to -1.9)	9.45(5.70 to 14.66)	2.42(1.26 to 4.27)	-4.33(-5.05 to -3.6)

Gambia	2892.79(2261.07 to 3419.69)	2326.95(1843.10 to 2736.62)	-0.7(-0.73 to -0.68)	9.68(5.32 to 15.58)	7.91(4.28 to 13.15)	-0.59(-0.88 to -0.31)
Georgia	58.55(39.16 to 85.10)	146.68(132.99 to 160.90)	3.04(2.83 to 3.24)	0.60(0.45 to 0.78)	0.07(0.05 to 0.10)	-6.22(-8.87 to -3.48)
Germany	150.97(98.62 to 223.73)	501.99(326.75 to 721.96)	3.96(3.69 to 4.23)	0.06(0.05 to 0.08)	0.02(0.01 to 0.03)	-3.35(-5.54 to -1.11)
Ghana	2848.86(2165.31 to 3465.44)	1817.20(1473.70 to 2104.60)	-1.69(-2.35 to -1.01)	3.74(2.07 to 6.18)	1.60(0.91 to 2.54)	-2.74(-3.1 to -2.39)
Greece	73.85(48.82 to 110.74)	247.12(156.00 to 372.32)	3.98(3.87 to 4.09)	0.02(0.02 to 0.03)	0.02(0.01 to 0.02)	-0.5(-2.33 to 1.37)
Greenland	71.01(45.84 to 107.66)	87.05(55.52 to 133.49)	0.66(0.62 to 0.71)	0.03(0.02 to 0.05)	0.01(0.01 to 0.02)	-2.81(-3.89 to -1.72)
Grenada	512.81(342.24 to 728.04)	347.19(237.31 to 486.62)	-1.25(-1.34 to -1.16)	0.89(0.66 to 1.13)	1.06(0.78 to 1.40)	0.6(-1.16 to 2.38)
Guam	506.81(335.53 to 704.78)	778.48(553.35 to 1010.38)	1.37(1.29 to 1.46)	0.20(0.11 to 0.31)	0.59(0.36 to 0.95)	3.97(2.65 to 5.31)
Guatemala	613.09(455.29 to 825.00)	185.73(140.98 to 249.11)	-3.77(-3.86 to -3.68)	4.93(3.51 to 6.71)	2.69(1.85 to 3.67)	-1.86(-6.93 to 3.49)
Guinea	3118.65(2478.54 to 3663.09)	2157.68(1700.96 to 2579.80)	-1.18(-1.2 to -1.16)	15.29(9.78 to 21.71)	9.67(5.55 to 16.06)	-1.48(-1.64 to -1.32)
Guinea-Bissau	3281.82(2571.59 to 3888.66)	2337.89(1817.91 to 2811.51)	-1.1(-1.12 to -1.07)	7.41(4.16 to 11.99)	3.85(2.01 to 6.92)	-2.06(-2.27 to -1.85)
Guyana	442.01(298.42 to 621.37)	350.01(240.00 to 491.19)	-0.75(-0.79 to -0.71)	1.27(0.85 to 1.78)	0.77(0.48 to 1.18)	-1.31(-3.04 to 0.45)
Haiti	1338.29(866.01 to 1956.30)	647.71(425.55 to 959.68)	-2.32(-2.39 to -2.25)	18.05(10.89 to 27.36)	11.48(6.31 to 18.86)	-1.36(-1.67 to -1.04)
Honduras	990.67(688.91 to 1443.55)	400.57(290.12 to 549.69)	-2.9(-3.17 to -2.63)	6.30(4.11 to 9.00)	2.11(1.03 to 3.74)	-3.24(-3.72 to -2.75)

Hungary	38.81(26.45 to 55.41)	122.51(79.49 to 185.75)	3.8(3.42 to 4.18)	0.07(0.05 to 0.10)	0.01(0.01 to 0.02)	-5.4(-7.39 to -3.38)
Iceland	136.69(87.62 to 209.69)	212.16(134.89 to 326.29)	1.41(1.24 to 1.58)	0.07(0.05 to 0.09)	0.02(0.01 to 0.03)	-3.71(-6.32 to -1.03)
India	673.16(466.23 to 930.76)	232.11(162.15 to 312.70)	-3.42(-3.56 to -3.28)	2.96(2.23 to 3.78)	1.24(0.91 to 1.64)	-2.7(-3.46 to -1.94)
Indonesia	700.05(478.99 to 954.26)	475.86(351.18 to 625.82)	-1.23(-1.32 to -1.14)	6.78(4.74 to 9.35)	2.26(1.58 to 3.14)	-3.46(-3.69 to -3.24)
Iran (Islamic Republic of)	1362.70(897.20 to 1882.28)	439.14(286.33 to 608.06)	-3.62(-3.8 to -3.44)	1.25(0.83 to 1.76)	0.05(0.03 to 0.07)	-9.98(-10.77 to -9.19)
Iraq	1686.64(1091.95 to 2427.82)	621.59(400.13 to 911.18)	-3.21(-3.31 to -3.12)	2.15(1.17 to 3.61)	0.41(0.22 to 0.71)	-5.24(-5.74 to -4.74)
Ireland	144.00(92.09 to 220.36)	257.53(160.61 to 390.09)	1.92(1.84 to 1.99)	0.06(0.04 to 0.07)	0.02(0.01 to 0.02)	-3.56(-5.8 to -1.27)
Israel	240.08(200.57 to 281.53)	442.64(273.58 to 681.16)	1.96(1.83 to 2.09)	0.05(0.04 to 0.07)	0.01(0.01 to 0.02)	-4.21(-5.84 to -2.56)
Italy	131.39(84.29 to 194.35)	190.87(148.99 to 245.49)	1.22(1.09 to 1.35)	0.04(0.04 to 0.05)	0.01(0.01 to 0.02)	-3.97(-8.74 to 1.05)
Jamaica	457.52(304.09 to 640.94)	330.45(228.03 to 453.59)	-1.06(-1.13 to -0.99)	0.80(0.58 to 1.09)	0.44(0.26 to 0.66)	-1.95(-4.76 to 0.95)
Japan	173.44(125.81 to 228.73)	444.35(343.95 to 555.04)	3.08(3.04 to 3.12)	0.05(0.03 to 0.06)	0.01(0.01 to 0.02)	-3.87(-4.56 to -3.17)
Jordan	592.47(375.90 to 903.52)	288.00(187.87 to 429.31)	-2.28(-2.48 to -2.09)	6.74(4.11 to 9.89)	0.17(0.08 to 0.30)	-11.34(-11.72 to -10.96)
Kazakhstan	331.26(219.34 to 460.25)	564.89(388.74 to 751.66)	1.78(1.62 to 1.94)	0.65(0.45 to 0.89)	0.15(0.10 to 0.20)	-4.8(-6 to -3.59)
Kenya	2429.03(1879.82 to 2920.10)	1208.70(949.65 to 1437.34)	-2.23(-2.27 to -2.19)	12.00(7.63 to 17.48)	4.82(2.84 to 7.67)	-2.9(-3.21 to -2.59)

Kiribati	993.34(663.04 to 1450.47)	683.40(446.48 to 971.98)	-1.19(-1.26 to -1.12)	2.25(1.26 to 3.68)	1.17(0.61 to 2.10)	-2.09(-2.39 to -1.8)
Kuwait	777.95(511.91 to 1096.08)	419.84(287.20 to 567.52)	-1.99(-2.11 to -1.87)	0.13(0.09 to 0.17)	0.02(0.01 to 0.03)	-6.19(-8.87 to -3.42)
Kyrgyzstan	319.07(201.05 to 477.49)	295.35(186.84 to 442.78)	-0.19(-0.38 to 0)	2.58(1.96 to 3.30)	0.35(0.25 to 0.47)	-6.4(-6.85 to -5.95)
Lao People's Democratic Republic	1446.50(964.22 to 2045.54)	416.61(285.28 to 577.48)	-3.95(-4.02 to -3.87)	8.22(4.66 to 13.03)	1.08(0.52 to 2.01)	-6.34(-6.49 to -6.2)
Latvia	142.32(87.43 to 212.54)	432.25(272.71 to 631.56)	3.81(3.44 to 4.19)	0.06(0.05 to 0.09)	0.01(0.00 to 0.01)	-8.06(-10.9 to -5.14)
Lebanon	562.14(359.94 to 825.37)	276.48(179.15 to 401.45)	-2.29(-2.37 to -2.21)	0.56(0.29 to 0.93)	0.05(0.03 to 0.09)	-7.1(-7.32 to -6.88)
Lesotho	1871.47(1341.02 to 2369.15)	870.23(652.07 to 1075.02)	-2.46(-2.5 to -2.42)	3.68(2.05 to 5.97)	3.62(1.73 to 6.75)	0.03(-1.08 to 1.16)
Liberia	3411.00(2706.32 to 3947.20)	2079.99(1661.62 to 2398.23)	-1.59(-1.63 to -1.56)	23.74(15.53 to 34.37)	1.65(0.88 to 2.74)	-8.21(-8.75 to -7.67)
Libya	1301.43(839.33 to 1868.05)	486.92(312.72 to 687.31)	-3.13(-3.19 to -3.07)	3.32(1.85 to 5.40)	0.52(0.27 to 0.93)	-5.81(-6.3 to -5.32)
Lithuania	166.64(105.98 to 249.24)	381.26(246.14 to 534.48)	2.72(2.1 to 3.35)	0.09(0.07 to 0.12)	0.02(0.01 to 0.02)	-5.2(-7.67 to -2.67)
Luxembourg	38.61(28.07 to 53.18)	101.16(68.33 to 148.36)	3.17(3.04 to 3.3)	0.07(0.05 to 0.09)	0.01(0.01 to 0.02)	-5.45(-7.53 to -3.31)
Madagascar	2699.06(2085.58 to 3220.00)	1415.36(1086.98 to 1690.07)	-2.07(-2.11 to -2.03)	9.91(6.18 to 14.81)	6.61(3.51 to 11.19)	-1.27(-1.63 to -0.9)
Malawi	3307.19(2575.70 to 3876.33)	1815.76(1432.38 to 2125.38)	-1.92(-1.94 to -1.89)	25.87(17.20 to 37.35)	8.54(4.71 to 14.06)	-3.49(-3.87 to -3.1)
Malaysia	877.49(575.02 to 1270.88)	734.19(505.51 to 996.34)	-0.56(-0.63 to -0.49)	0.96(0.56 to 1.51)	0.38(0.22 to 0.60)	-2.95(-3.78 to -2.11)

Maldives	1311.04(857.07 to 1914.61)	431.25(281.76 to 611.36)	-3.51(-3.59 to -3.43)	2.71(1.49 to 4.31)	0.13(0.08 to 0.22)	-9.45(-10.2 to -8.7)
Mali	3403.51(2631.22 to 4052.45)	2896.34(2231.92 to 3479.55)	-0.52(-0.53 to -0.5)	6.80(4.51 to 9.97)	2.93(1.78 to 4.58)	-2.7(-3.08 to -2.32)
Malta	167.15(104.30 to 250.04)	247.31(150.52 to 377.05)	1.59(-0.17 to 3.38)	0.08(0.06 to 0.11)	0.03(0.02 to 0.04)	-3.48(-5.42 to -1.49)
Marshall Islands	559.22(359.96 to 826.07)	405.44(266.64 to 590.85)	-1.02(-1.06 to -0.97)	0.85(0.41 to 1.69)	0.16(0.08 to 0.30)	-5.3(-7.45 to -3.1)
Mauritania	3420.05(2739.28 to 3930.17)	2070.61(1702.24 to 2369.72)	-1.61(-1.65 to -1.57)	27.02(18.63 to 37.84)	5.67(2.73 to 10.39)	-4.9(-5.12 to -4.69)
Mauritius	300.96(198.33 to 430.81)	294.26(197.74 to 406.64)	-0.06(-0.12 to 0.01)	0.61(0.45 to 0.83)	0.28(0.21 to 0.37)	-2.54(-4.24 to -0.81)
Mexico	1173.65(929.80 to 1425.65)	561.32(479.85 to 628.24)	-2.35(-2.41 to -2.3)	1.41(1.04 to 1.83)	0.26(0.18 to 0.36)	-5.21(-5.89 to -4.53)
Micronesia (Federated States of)	1280.82(841.50 to 1857.64)	699.58(471.91 to 989.67)	-1.93(-1.95 to -1.91)	1.90(0.97 to 3.36)	0.29(0.14 to 0.54)	-5.91(-6.41 to -5.42)
Monaco	164.25(103.81 to 247.94)	287.41(177.76 to 433.01)	1.83(1.79 to 1.87)	0.04(0.01 to 0.07)	0.03(0.01 to 0.05)	-1.41(-1.56 to -1.26)
Mongolia	473.92(289.32 to 709.38)	405.48(257.93 to 598.68)	-0.42(-0.69 to -0.15)	1.56(0.93 to 2.43)	0.30(0.17 to 0.46)	-5.08(-5.52 to -4.64)
Montenegro	145.76(92.22 to 221.16)	217.37(134.66 to 323.80)	1.34(1.23 to 1.44)	0.01(0.01 to 0.02)	0.00(0.00 to 0.01)	-3.64(-4.32 to -2.95)
Morocco	1189.97(790.01 to 1716.94)	591.79(389.89 to 840.66)	-2.24(-2.28 to -2.21)	12.80(8.64 to 18.53)	1.19(0.58 to 2.09)	-7.35(-7.7 to -7)
Mozambique	2787.36(2150.21 to 3332.70)	1945.07(1535.64 to 2280.34)	-1.16(-1.18 to -1.13)	13.68(8.66 to 19.92)	7.52(3.67 to 13.47)	-1.9(-2.25 to -1.55)

Myanmar	918.85(605.75 to 1328.35)	553.07(366.45 to 798.56)	-1.62(-1.66 to -1.58)	1.39(0.86 to 2.11)	1.63(0.93 to 2.70)	0.52(0.04 to 0.99)
Namibia	2211.19(1596.50 to 2777.21)	1021.38(750.93 to 1292.31)	-2.47(-2.55 to -2.38)	5.69(3.52 to 8.73)	1.63(0.77 to 3.08)	-3.96(-4.44 to -3.48)
Nauru	1107.20(747.20 to 1566.36)	683.70(464.13 to 948.19)	-1.56(-1.63 to -1.49)	4.34(2.19 to 7.76)	3.68(1.79 to 6.81)	-0.53(-0.64 to -0.42)
Nepal	966.50(627.76 to 1396.87)	149.86(95.88 to 214.64)	-5.88(-6.04 to -5.72)	10.22(5.72 to 16.13)	1.12(0.59 to 2.00)	-6.92(-7.52 to -6.3)
Netherlands	75.91(49.61 to 115.40)	163.14(104.92 to 250.50)	2.4(1.84 to 2.96)	0.09(0.07 to 0.12)	0.02(0.01 to 0.03)	-4.8(-5.6 to -3.99)
New Zealand	316.36(219.50 to 428.85)	443.89(331.80 to 586.65)	1.09(0.97 to 1.21)	0.03(0.02 to 0.03)	0.01(0.01 to 0.02)	-2.89(-6.57 to 0.95)
Nicaragua	634.61(442.67 to 898.53)	289.39(211.53 to 393.17)	-2.5(-2.66 to -2.35)	1.75(1.16 to 2.49)	0.37(0.22 to 0.57)	-4.99(-5.18 to -4.81)
Niger	3394.91(2609.71 to 4054.99)	3100.89(2292.01 to 3787.33)	-0.29(-0.31 to -0.26)	12.21(8.00 to 18.42)	8.19(4.66 to 13.26)	-1.22(-1.56 to -0.88)
Nigeria	3145.55(2462.41 to 3709.09)	2173.99(1732.55 to 2562.21)	-1.19(-1.3 to -1.08)	6.31(3.68 to 9.66)	8.97(5.47 to 14.02)	1.16(0.97 to 1.35)
Niue	630.65(407.30 to 936.57)	560.71(386.29 to 753.97)	-0.37(-0.41 to -0.33)	0.89(0.41 to 1.64)	0.32(0.17 to 0.54)	-3.31(-3.93 to -2.69)
North Macedonia	72.12(46.57 to 108.99)	131.77(82.22 to 203.85)	1.99(1.88 to 2.09)	0.12(0.07 to 0.18)	0.02(0.01 to 0.03)	-5.7(-7.13 to -4.24)
Northern Mariana Islands	559.45(387.93 to 744.64)	523.05(376.03 to 685.00)	-0.22(-0.24 to -0.19)	0.71(0.35 to 1.23)	0.48(0.26 to 0.80)	-1.07(-2.65 to 0.54)
Norway	229.48(154.87 to 321.10)	393.99(257.97 to 562.52)	1.66(0.92 to 2.39)	0.09(0.07 to 0.11)	0.01(0.01 to 0.01)	-8.76(-11.85 to -5.57)
Oman	1440.22(897.04 to 2126.81)	755.09(498.24 to 1068.67)	-2.05(-2.1 to -2)	2.00(0.98 to 3.75)	0.23(0.13 to 0.41)	-6.76(-7.49 to -6.02)

Pakistan	1773.78(1191.34 to 2477.86)	722.18(484.79 to 991.03)	-2.86(-2.95 to -2.77)	12.34(7.32 to 19.51)	7.76(4.00 to 13.06)	-1.4(-1.76 to -1.03)
Palau	565.08(374.45 to 819.62)	537.05(365.15 to 721.39)	-0.16(-0.21 to -0.1)	0.89(0.44 to 1.57)	0.42(0.22 to 0.74)	-2.51(-2.91 to -2.1)
Palestine	1033.52(643.46 to 1585.16)	336.56(210.45 to 504.19)	-3.59(-3.71 to -3.46)	0.89(0.44 to 1.52)	0.13(0.07 to 0.22)	-5.85(-6.66 to -5.04)
Panama	322.17(230.70 to 450.98)	328.93(239.54 to 450.04)	0.1(-0.1 to 0.31)	0.81(0.60 to 1.05)	0.37(0.24 to 0.51)	-2.58(-3.79 to -1.35)
Papua New Guinea	1065.10(698.53 to 1568.18)	787.22(524.24 to 1134.23)	-0.98(-1.02 to -0.94)	2.48(1.14 to 4.34)	1.69(0.96 to 2.80)	-1.17(-1.67 to -0.66)
Paraguay	778.05(526.11 to 1136.02)	497.88(336.27 to 710.28)	-1.41(-1.45 to -1.37)	2.48(1.66 to 3.53)	0.84(0.49 to 1.36)	-3.23(-4.2 to -2.26)
Peru	289.89(198.01 to 416.55)	160.72(118.53 to 220.91)	-1.94(-2.35 to -1.53)	4.26(2.62 to 6.24)	1.13(0.61 to 1.89)	-4.11(-5.61 to -2.59)
Philippines	1132.55(757.89 to 1577.37)	620.42(417.63 to 859.63)	-1.93(-1.97 to -1.88)	2.92(1.94 to 4.05)	1.65(1.13 to 2.39)	-1.74(-2.11 to -1.37)
Poland	109.82(65.23 to 170.94)	114.88(89.89 to 147.30)	0.24(-0.21 to 0.7)	0.05(0.03 to 0.06)	0.00(0.00 to 0.00)	-8.91(-10.5 to -7.3)
Portugal	77.91(48.34 to 119.44)	205.80(189.81 to 221.26)	3.14(2.88 to 3.4)	0.06(0.05 to 0.08)	0.03(0.02 to 0.04)	-2.48(-4.57 to -0.34)
Puerto Rico	264.70(187.88 to 354.59)	187.73(141.81 to 243.10)	-1.09(-1.3 to -0.89)	0.36(0.27 to 0.45)	0.11(0.08 to 0.16)	-3.74(-5.6 to -1.83)
Qatar	742.61(471.56 to 1078.78)	526.77(352.68 to 718.97)	-1.09(-1.29 to -0.9)	0.47(0.26 to 0.79)	0.02(0.01 to 0.03)	-10.38(-11.9 to -8.84)
Republic of Korea	18.06(14.53 to 22.72)	73.29(53.35 to 100.29)	4.53(4.13 to 4.93)	0.12(0.08 to 0.17)	0.02(0.01 to 0.03)	-5.98(-6.36 to -5.6)
Republic of Moldova	155.39(96.42 to 235.79)	171.30(106.50 to 253.23)	0.33(0.19 to 0.48)	0.17(0.13 to 0.23)	0.01(0.01 to 0.01)	-8.55(-11.55 to -5.45)

Romania	111.46(72.21 to 165.55)	268.28(172.37 to 390.37)	2.93(2.73 to 3.12)	0.08(0.06 to 0.12)	0.04(0.03 to 0.05)	-1.92(-4.51 to 0.74)
Russian Federation	133.08(86.35 to 191.84)	369.53(248.13 to 500.07)	3.41(3.11 to 3.71)	0.14(0.11 to 0.18)	0.02(0.01 to 0.03)	-6.13(-6.63 to -5.62)
Rwanda	4013.69(3116.35 to 4738.33)	1629.40(1264.18 to 1929.62)	-2.88(-2.93 to -2.83)	55.43(35.66 to 82.31)	11.64(6.65 to 18.59)	-4.83(-5.13 to -4.53)
Saint Kitts and Nevis	408.10(271.36 to 577.83)	225.63(157.87 to 309.74)	-1.9(-2.05 to -1.75)	4.45(3.45 to 5.68)	1.32(0.87 to 1.90)	-3.75(-4.66 to -2.82)
Saint Lucia	560.08(368.83 to 796.52)	286.57(194.23 to 399.46)	-2.14(-2.18 to -2.09)	0.57(0.41 to 0.77)	0.17(0.11 to 0.24)	-4.15(-4.9 to -3.4)
Saint Vincent and the Grenadines	419.23(279.17 to 596.01)	391.65(264.56 to 553.56)	-0.23(-0.34 to -0.12)	1.07(0.83 to 1.34)	0.61(0.43 to 0.82)	-1.97-2.86 to -1.07)
Samoa	710.61(480.80 to 990.54)	1047.91(694.37 to 1452.93)	1.28(1.21 to 1.34)	0.38(0.19 to 0.66)	0.24(0.12 to 0.44)	-1.48(-1.85 to -1.1)
San Marino	120.79(76.57 to 181.95)	301.29(190.16 to 460.57)	3(2.94 to 3.07)	0.02(0.01 to 0.04)	0.01(0.00 to 0.01)	-3.54(-3.77 to -3.31)
Sao Tome and Principe	2794.58(2191.91 to 3255.63)	1102.46(899.08 to 1268.21)	-2.98(-3.08 to -2.87)	6.24(3.53 to 9.79)	1.38(0.68 to 2.57)	-4.72(-5.47 to -3.96)
Saudi Arabia	1958.78(1263.98 to 2804.30)	535.58(367.40 to 726.84)	-4.1(-4.21 to -4)	2.49(1.25 to 4.31)	0.24(0.12 to 0.43)	-7.28(-7.54 to -7.01)
Senegal	3685.85(2927.63 to 4301.99)	2030.84(1612.96 to 2390.11)	-1.91(-1.95 to -1.87)	14.97(9.48 to 21.92)	4.70(2.66 to 7.38)	-3.7(-4.01 to -3.38)
Serbia	62.42(40.70 to 93.10)	123.23(78.49 to 190.97)	2.22(2.06 to 2.38)	0.17(0.10 to 0.25)	0.02(0.01 to 0.04)	-6.19(-7.65 to -4.72)
Seychelles	467.66(310.82 to 673.42)	538.98(371.58 to 736.95)	0.46(0.39 to 0.53)	3.05(1.90 to 4.48)	2.07(1.31 to 3.05)	-1.39(-2.12 to -0.65)
Sierra Leone	2808.86(2187.04 to 3330.62)	1802.31(1378.92 to 2154.95)	-1.43(-1.45 to -1.4)	5.47(2.88 to 9.11)	3.81(1.95 to 6.47)	-1.04(-1.59 to -0.49)

Singapore	113.58(70.99 to 174.96)	149.95(92.31 to 229.10)	0.86(0.72 to 1.01)	0.03(0.02 to 0.05)	0.00(0.00 to 0.01)	-6.64(-8.91 to -4.32)
Slovakia	59.83(38.62 to 88.83)	167.63(102.20 to 252.70)	3.32(2.77 to 3.88)	0.05(0.03 to 0.07)	0.01(0.00 to 0.01)	-5.85(-6.34 to -5.36)
Slovenia	76.18(49.43 to 111.03)	299.19(184.55 to 441.54)	4.58(4.41 to 4.75)	0.04(0.03 to 0.05)	0.00(0.00 to 0.00)	-8.21(-9.97 to -6.4)
Solomon Islands	1399.79(927.88 to 2063.01)	818.61(539.54 to 1190.25)	-1.72(-1.74 to -1.7)	3.14(1.09 to 6.22)	2.36(1.29 to 3.95)	-0.96(-1.21 to -0.71)
Somalia	3494.21(2694.62 to 4181.98)	2651.11(1986.74 to 3264.64)	-0.88(-0.9 to -0.86)	14.98(8.49 to 23.89)	6.42(3.48 to 10.94)	-2.72(-2.93 to -2.52)
South Africa	1164.35(880.87 to 1411.26)	578.66(452.27 to 692.00)	-2.23(-2.27 to -2.18)	3.81(2.45 to 5.58)	1.44(0.95 to 1.97)	-3.14(-4.51 to -1.75)
South Sudan	3680.01(2930.16 to 4282.68)	3404.51(2744.38 to 3945.33)	-0.25(-0.26 to -0.24)	15.45(7.86 to 26.37)	13.35(7.32 to 22.70)	-0.47(-0.78 to -0.16)
Spain	177.95(117.85 to 262.63)	368.28(357.65 to 379.97)	2.42(2.07 to 2.77)	0.03(0.02 to 0.04)	0.01(0.01 to 0.02)	-1.85(-2.69 to -0.99)
Sri Lanka	421.22(276.66 to 598.81)	434.24(289.72 to 622.72)	0.11(0.03 to 0.18)	0.54(0.33 to 0.83)	0.11(0.05 to 0.19)	-4.91(-5.96 to -3.85)
Sudan	1308.13(855.30 to 1926.76)	540.39(341.24 to 788.35)	-2.83(-2.92 to -2.75)	23.35(14.94 to 36.09)	4.05(1.94 to 7.44)	-5.49(-5.7 to -5.28)
Suriname	400.43(268.67 to 569.67)	344.74(234.41 to 491.13)	-0.47(-0.58 to -0.36)	1.35(0.76 to 2.18)	0.83(0.47 to 1.35)	-1.66(-2.99 to -0.32)
Sweden	106.41(77.46 to 143.45)	213.76(134.68 to 318.92)	2.22(2.05 to 2.38)	0.04(0.03 to 0.05)	0.01(0.01 to 0.02)	-1.85(-3 to -0.7)
Switzerland	103.96(65.29 to 160.78)	272.35(168.74 to 419.98)	3.17(2.95 to 3.39)	0.04(0.03 to 0.06)	0.01(0.01 to 0.01)	-5.37(-6.08 to -4.66)
Syrian Arab Republic	1189.99(756.94 to 1717.06)	480.19(311.08 to 704.40)	-2.88(-2.92 to -2.83)	2.59(1.49 to 4.12)	0.18(0.09 to 0.33)	-8.29(-9.84 to -6.71)

Taiwan (Province of China)	40.63(26.70 to 59.81)	133.44(84.37 to 197.38)	4.07(3.46 to 4.68)	0.05(0.04 to 0.07)	0.02(0.01 to 0.03)	-3(-5.37 to -0.57)
Tajikistan	489.66(306.59 to 741.69)	196.94(124.10 to 293.33)	-2.88(-2.95 to -2.81)	1.81(1.20 to 2.58)	0.31(0.17 to 0.52)	-5.49(-6 to -4.98)
Thailand	292.52(196.80 to 418.22)	245.66(166.96 to 342.73)	-0.56(-0.6 to -0.51)	0.20(0.10 to 0.35)	0.20(0.12 to 0.33)	0.15(-0.57 to 0.88)
Timor-Leste	1698.09(1148.67 to 2394.14)	917.74(614.94 to 1315.66)	-1.97(-2 to -1.95)	15.84(8.87 to 24.74)	2.75(1.53 to 4.68)	-5.56(-6.23 to -4.88)
Togo	3334.94(2577.36 to 4009.28)	1939.81(1494.12 to 2373.35)	-1.74(-1.8 to -1.67)	9.07(5.55 to 13.74)	2.42(1.28 to 4.06)	-4.12(-4.5 to -3.74)
Tokelau	705.16(452.14 to 1041.17)	603.26(404.93 to 860.22)	-0.51(-0.53 to -0.49)	0.82(0.38 to 1.62)	0.38(0.20 to 0.66)	-2.47(-2.57 to -2.38)
Tonga	1735.08(1142.98 to 2444.99)	1043.11(718.02 to 1400.81)	-1.63(-1.66 to -1.59)	0.98(0.53 to 1.69)	0.51(0.25 to 0.92)	-2.03(-2.24 to -1.83)
Trinidad and Tobago	437.97(300.41 to 600.87)	361.28(256.45 to 486.52)	-0.63(-0.82 to -0.44)	0.86(0.62 to 1.13)	0.64(0.43 to 0.94)	-0.9(-1.98 to 0.2)
Tunisia	735.62(466.24 to 1084.27)	395.65(254.73 to 581.91)	-1.96(-2.04 to -1.87)	1.55(0.92 to 2.41)	0.22(0.11 to 0.41)	-6.12(-6.44 to -5.79)
Turkey	472.17(314.17 to 669.92)	407.00(274.63 to 555.77)	-0.45(-0.78 to -0.12)	3.69(2.15 to 5.64)	0.14(0.08 to 0.23)	-9.8(-10.78 to -8.8)
Turkmenistan	513.06(322.87 to 773.06)	278.72(178.44 to 405.93)	-1.94(-2.07 to -1.82)	1.26(0.88 to 1.71)	0.65(0.42 to 0.97)	-2.13(-2.75 to -1.5)
Tuvalu	1122.57(737.48 to 1643.43)	701.16(469.61 to 996.47)	-1.51(-1.57 to -1.44)	1.79(0.81 to 3.64)	0.86(0.43 to 1.58)	-2.36(-2.58 to -2.14)
Uganda	3121.09(2412.17 to 3716.99)	1901.62(1494.29 to 2243.73)	-1.58(-1.61 to -1.55)	25.84(15.79 to 39.39)	8.66(4.74 to 14.16)	-3.48(-3.75 to -3.22)
Ukraine	114.52(73.54 to 163.78)	218.81(144.81 to 309.38)	2.15(1.85 to 2.46)	0.11(0.08 to 0.15)	0.01(0.01 to 0.02)	-6.75(-8.61 to -4.85)

United Arab Emirates	876.70(689.94 to 1078.07)	535.57(353.92 to 740.61)	-1.57(-1.63 to -1.5)	0.73(0.33 to 1.42)	0.08(0.04 to 0.14)	-6.9(-7.64 to -6.16)
United Kingdom	135.88(91.57 to 194.34)	337.09(215.65 to 495.65)	2.97(2.91 to 3.02)	0.05(0.04 to 0.06)	0.01(0.01 to 0.01)	-4.65(-5.5 to -3.79)
United Republic of Tanzania	3023.87(2351.43 to 3547.21)	1879.77(1483.85 to 2210.28)	-1.51(-1.6 to -1.43)	9.86(6.43 to 14.20)	8.61(5.11 to 13.47)	-0.42(-0.67 to -0.17)
United States of America	216.34(151.16 to 304.79)	498.24(388.34 to 627.35)	2.7(2.62 to 2.78)	0.06(0.05 to 0.09)	0.09(0.06 to 0.13)	1.33(0.46 to 2.21)
United States Virgin Islands	435.03(298.04 to 592.21)	477.59(355.49 to 624.44)	0.3(0.26 to 0.34)	1.02(0.58 to 1.66)	0.21(0.11 to 0.36)	-5.43(-5.94 to -4.92)
Uruguay	378.83(242.93 to 560.92)	470.49(305.26 to 666.58)	0.7(0.63 to 0.76)	0.45(0.33 to 0.59)	0.12(0.09 to 0.17)	-4.1(-5.94 to -2.22)
Uzbekistan	339.00(210.48 to 510.93)	166.89(105.49 to 248.65)	-2.27(-2.35 to -2.2)	1.17(0.85 to 1.54)	0.21(0.14 to 0.29)	-5.57(-6.89 to -4.22)
Vanuatu	1205.03(790.98 to 1755.89)	694.13(462.21 to 997.36)	-1.78(-1.82 to -1.75)	2.11(0.93 to 4.33)	1.93(1.01 to 3.41)	-0.27(-0.69 to 0.16)
Venezuela (Bolivarian Republic of)	557.30(389.59 to 769.07)	319.42(234.41 to 433.10)	-1.76(-1.88 to -1.64)	1.58(1.22 to 2.00)	0.71(0.46 to 1.03)	-1.9(-2.45 to -1.35)
Viet Nam	510.25(346.42 to 733.13)	232.52(154.56 to 332.24)	-2.52(-2.63 to -2.42)	0.12(0.05 to 0.23)	0.03(0.02 to 0.06)	-4.38(-4.46 to -4.31)
Yemen	3203.64(2304.59 to 4061.01)	1379.08(951.94 to 1851.88)	-2.69(-2.73 to -2.66)	8.63(4.72 to 13.72)	2.33(1.18 to 4.03)	-4.15(-4.33 to -3.97)
Zambia	3334.70(2659.51 to 3860.84)	1969.11(1611.89 to 2252.41)	-1.68(-1.71 to -1.65)	6.21(3.84 to 9.48)	2.75(1.45 to 4.66)	-2.58(-2.85 to -2.32)
Zimbabwe	2015.21(1458.89 to 2538.83)	1104.46(776.49 to 1438.59)	-1.92(-1.98 to -1.86)	4.44(2.66 to 6.81)	5.28(2.75 to 9.26)	0.8(0.02 to 1.59)

ASIR, age-standardized incidence rate. ASDR, age-standardized death rate. AAPC, average annual percent change.

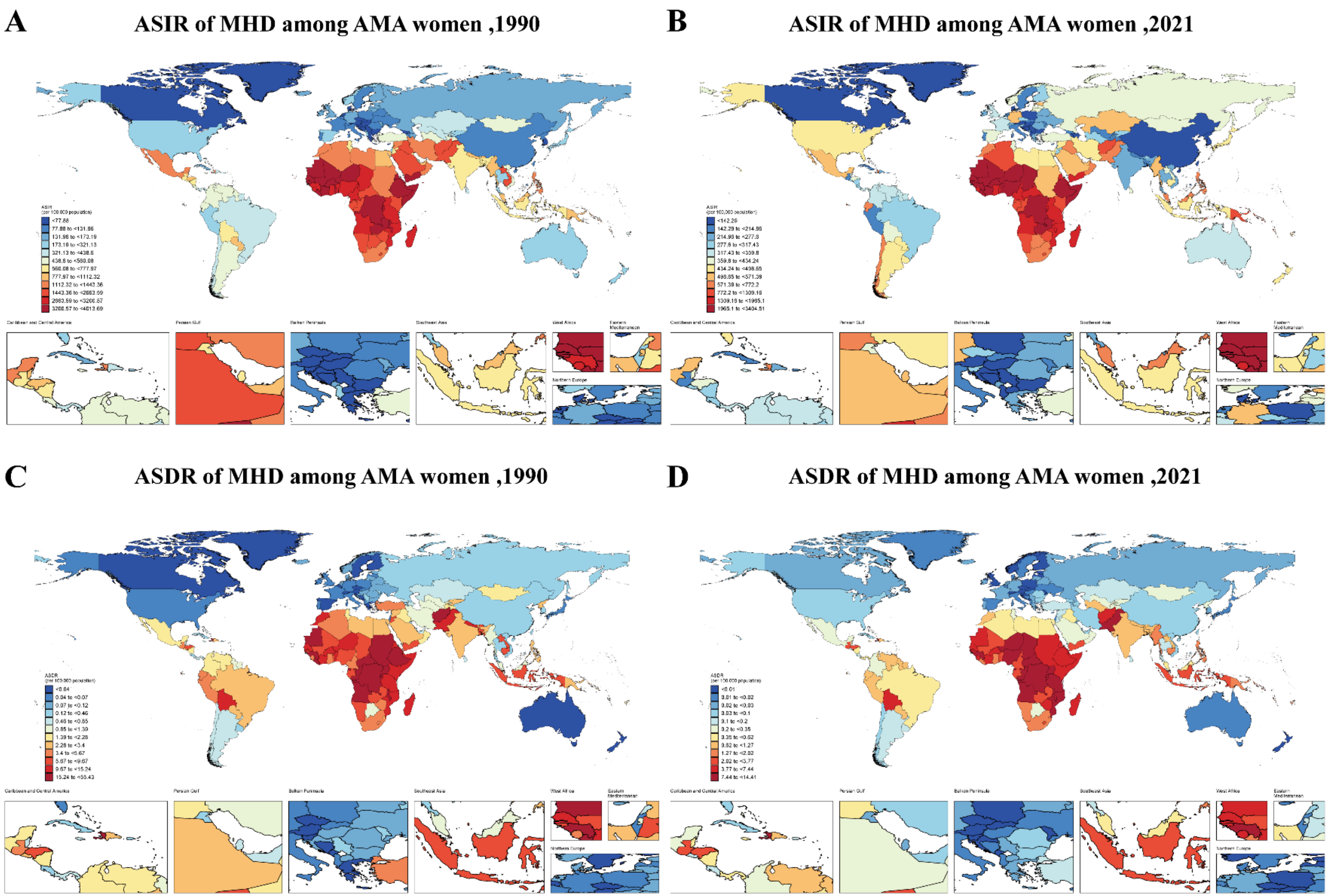

Fig.S1 ASIR and ASDR of maternal hypertensive disorders among women of AMA across all countries in 1990 and 2021. (A) ASIR in 1990; (B)ASIR in 2021;(C) ASDR in 1990; (D) ASDR in 2021. ASIR, age-standardized incidence rate; MHD, maternal hypertensive disorders. ASDR, age-standardized death rate; AMA, advanced maternal age. Map lines delineate study areas and do not necessarily depict accepted national boundaries

Supplementary table 3: Regional Hierarchy and Country Classification in the GBD 2021 Study

GBD Region	Countries and Territories
Central Asia	Armenia, Azerbaijan, Georgia, Kazakhstan, Kyrgyzstan, Mongolia, Tajikistan, Turkmenistan, Uzbekistan
Central Europe	Albania, Bosnia and Herzegovina, Bulgaria, Croatia, Czech Republic, Hungary, North Macedonia, Montenegro, Poland, Romania, Serbia, Slovakia, Slovenia
Eastern Europe	Belarus, Estonia, Latvia, Lithuania, Moldova, Russia, Ukraine
Australasia	Australia, New Zealand
High-income Asia Pacific	Brunei, Japan, Singapore, South Korea
High-income North America	Canada, United States， Greenland
Southern Latin America	Argentina, Chile, Uruguay
Western Europe	Andorra, Austria, Belgium, Cyprus, Denmark, Finland, France, Germany, Greece, Iceland, Ireland, Israel, Italy, Luxembourg, Malta, Monaco, Netherlands, Norway, Portugal, Spain, San Marino, Sweden, Switzerland, United Kingdom
Andean Latin America	Bolivia, Ecuador, Peru
Caribbean	Antigua and Barbuda, The Bahamas, Barbados, Belize, Bermuda, Cuba, Dominica, Dominican Republic, Grenada, Guyana, Haiti, Jamaica, Puerto Rico, Saint Kitts and Nevis, Saint Lucia, Saint Vincent and the Grenadines, Suriname, Trinidad and Tobago, United States Virgin Islands
Central Latin America	Colombia, Costa Rica, El Salvador, Guatemala, Honduras, Mexico, Nicaragua, Panama, Venezuela
Tropical Latin America	Brazil, Paraguay
North Africa and Middle East	Afghanistan, Algeria, Bahrain, Egypt, Iran, Iraq, Jordan, Kuwait, Lebanon, Libya, Morocco, Oman, Palestine, Qatar, Saudi Arabia, Sudan, Syria, Tunisia, Turkey, United Arab Emirates, Yemen
South Asia	Bangladesh, Bhutan, India, Nepal, Pakistan
East Asia	China, North Korea, Taiwan (Province of China)
Oceania	American Samoa, Federated States of Micronesia, Fiji, Guam, Kiribati, Marshall Islands, Northern Mariana Islands, Papua New Guinea, Samoa, Solomon Islands, Tonga, Vanuatu
Southeast Asia	Cambodia, Indonesia, Laos, Malaysia, Maldives, Mauritius, Myanmar, Philippines, Seychelles, Sri Lanka, Thailand, Timor-Leste, Vietnam
Central Sub-Saharan Africa	Angola, Central African Republic, Congo, Democratic Republic of the Congo, Equatorial Guinea, Gabon
Eastern Sub-Saharan Africa	Burundi, Comoros, Djibouti, Eritrea, Ethiopia, Kenya, Madagascar, Malawi, Mozambique, Rwanda, Somalia, South Sudan, Sudan, Tanzania, Uganda, Zambia
Southern Sub-Saharan Africa	Botswana, Eswatini, Lesotho, Namibia, South Africa, Zimbabwe
Western Sub-Saharan Africa	Benin, Burkina Faso, Cameroon, Cape Verde, Chad, Côte d'Ivoire, The Gambia, Ghana, Guinea, Guinea-Bissau, Liberia, Mali, Mauritania, Niger, Nigeria, São Tomé and Príncipe, Senegal, Sierra Leone, Togo
